# An intervention to support stroke survivors and their carers in the longer term: results of a cluster randomised controlled feasibility trial (LoTS2Care)

**DOI:** 10.1186/s40814-023-01258-6

**Published:** 2023-03-15

**Authors:** Anne Forster, Seline Ozer, Richard Brindle, Lorna Barnard, Natasha Hardicre, Thomas F. Crocker, Marie Chenery, Lauren Moreau, Alan Wright, Louisa-Jane Burton, Suzanne Hartley, Claire Hulme, Bryony Dawkins, Ivana Holloway, Allan House, Jenny Hewison, Amanda Farrin, on behalf of the LoTS2Care Programme Team, Ross Atkinson, Arvin Prashar, Nicola Cornwall, David Clarke, David Meads, Laetitia Schmitt, John Young, Josie Dickerson, Rebecca Hawkins, Rosemary McEachan, Matthew Fay, Christopher McKevitt, Robbie Foy, Gillian Carter, Gillian Richardson, Elaine Roberts

**Affiliations:** 1 Academic Unit for Ageing and Stroke Research, Leeds Institute of Health Sciences, University of Leeds, Leeds, UK; 2 Academic Unit for Ageing and Stroke Research, Bradford Institute for Health Research, Bradford Teaching Hospitals NHS Foundation Trust, Bradford, UK; 3 Clinical Trials Research Unit, Leeds Institute of Clinical Trials Research, University of Leeds, Leeds, UK; 4 College of Medicine and Health, University of Exeter, Exeter, UK; 5 Academic Unit of Health Economics, University of Leeds, Leeds, UK; 6 Leeds Institute of Health Sciences, University of Leeds, Leeds, UK; 7 Division of Health Services Research, Leeds Institute of Health Sciences, University of Leeds, Leeds, UK

**Keywords:** Stroke, Longer term, Feasibility trial, Community, Quality of life, Activities of daily living, Health services research, Cluster randomised controlled trial, Supported self-management

## Abstract

**Background:**

To address the limited provision of longer-term stroke care, we conducted a programme of research (LoTS2Care) to develop and test an intervention to form part of a replicable longer-term care strategy. New Start, a programme of facilitated self-management, was developed to be delivered at 6 months post-stroke by trained facilitators. Here, we report the findings from the final workstream of this programme, which aimed to evaluate the feasibility and acceptability of implementing a future definitive cluster randomised controlled trial of the developed intervention (New Start) to support stroke survivors and their carers in the longer term.

**Methods:**

A feasibility cluster randomised controlled trial was conducted in English and Welsh NHS stroke services. Stroke services (clusters) were randomised on a 1:1 basis to implement New Start or continue with usual care only. Community-dwelling stroke survivors between 4 and 6 months post-stroke were invited to participate in the trial by post. Outcome measures were collected via post at 3, 6 and 9 months after recruitment. Recruitment and follow-up rates, delivery and uptake of the intervention, data collection feasibility (including postal outcome measures of health and disability, mental well-being at 3, 6, and 9 months post-recruitment) and safety were assessed.

**Results:**

Ten stroke services were recruited. A total of 1127 stroke survivors were screened for participation, and 269 were registered (New Start, *n* = 145; usual care, *n* = 124). Retention was high with 239 (89%) stroke survivors being available for follow-up at 9 months, and high return rates of postal questionnaires were achieved (80.3% at 9 months). Intervention training was successfully delivered, and New Start was offered to 95.2% of trial participants in the intervention arm. Uptake was variable, however, ranging from 11.8 to 75.0%. There were no safety concerns.

**Conclusions:**

Stroke service recruitment and longer-term stroke survivor postal recruitment and outcome data collection are feasible; however, refinement of intervention targeting and delivery is required prior to undertaking a definitive trial.

**Trial registration:**

ISRCTN38920246. Registered 22 June 2016 (http://www.isrctn.com/ISRCTN38920246).

**Supplementary Information:**

The online version contains supplementary material available at 10.1186/s40814-023-01258-6.

## Key messages regarding feasibility

- Uncertainties regarding feasibility: There are limited opportunities to identify and engage with stroke survivors once provision of stroke care services is ended. A key aim of this feasibility trial therefore was to assess the feasibility of recruiting longer-term stroke survivors post-discharge. There were also uncertainties around the feasibility of implementing the intervention, including recruiting and training facilitators to deliver the New Start intervention, and around the acceptability of the intervention. In addition, there were uncertainties around the appropriateness of the outcome measures and so follow-up and completion rates were assessed to inform the choice of primary and secondary outcomes for the definitive trial.
- Key findings: Postal recruitment of longer-term stroke survivors was feasible, and retention was high with 89% participants being available for follow-up at 9 months. High return rates of postal questionnaires were achieved (80.3% of recruited participants at 9 months); however, few of the 36-item World Health Organization Disability Assessment Schedule (WHODAS) 2.0 questionnaires were fully completed (20% of available participants), although flexibility in the scoring system meant that 73.6% of available participants could still be scored using the simple score. There were higher completion rates for the shorter 14-item Warwick-Edinburgh Mental Well-being Scale (WEMWBS) (77.8% of questionnaires from available participants were fully completed; 83.7% were able to be scored). In all intervention services, facilitators were appointed, successfully trained and went on to deliver New Start; however, uptake by stroke survivors varied between services.
- Implications of the findings: The trial demonstrated that postal recruitment and follow-up are feasible and acceptable to longer-term stroke survivors. Unsurprisingly, length of questionnaire appeared to have an influence on completion rates, with higher completion rates for the WEMWBS questionnaire (14 items) than the WHODAS (36 items). In addition, New Start requires further refinement and for the target population to be more clearly specified, for example through screening for unmet need, prior to evaluation in a definitive trial.

## Background

Stroke remains a major illness and is one of the leading causes of adult disability in the UK. There are over 1.2 million stroke survivors in the UK, and over 100,000 people suffer a stroke each year [1]. Care of patients after stroke has been transformed, with the establishment of the recommended stroke care pathway in the first weeks after stroke [2]. However, despite policy recommendations, strategies for longer-term care are not developed, and the provision of posthospital rehabilitation and support remains varied [3]. As a result, longer-term outcomes are often poor [4–7] with many survivors reporting unmet needs long after their stroke. These needs are multi-faceted and influenced by a range of social and environmental factors. One survey of 1251 stroke survivors identified a variety of commonly reported needs, including those related to information provision (54%), mobility problems (25%), falls (21%), incontinence (21%), pain (15%) and fatigue (43%) [8].

Stroke survivors often rely on help from informal carers to assist with activities of daily living (ADL) [9], and the burden of caregiving can have an impact on carers’ physical and psychosocial well-being [10, 11], with up to 48% of carers reporting health problems and two-thirds reporting a decline in social life, with high self-reported levels of strain [12].

To address the limited provision of longer-term stroke care, we conducted a programme of research (LoTS2Care) to develop and test an intervention to form part of a replicable longer-term care strategy. The intent was to improve the quality of life (QoL) of stroke survivors and their carers by addressing unmet needs and enhancing participation (i.e. involvement in life situations). The intervention developed through this programme, called New Start, includes key components such as problem-solving self-management with stroke survivors and carers, which help with obtaining useable information and building sustainable flexible support networks, aiming to address needs at 6 months post-stroke. New Start was designed to be offered routinely to all stroke survivors at 6 months post-stroke in keeping with guidance for review at that time point. This paper reports findings from the final workstream of the LoTS2Care programme: a cluster randomised controlled feasibility trial (cRCT) of New Start. An embedded process evaluation exploring intervention fidelity and implementation and exploratory cost-effectiveness evaluation of the intervention are reported elsewhere [13]. A full list of the feasibility trial objectives can be found in Appendix 1. Here, we focus on the key objectives related to evaluating the feasibility and acceptability of implementing a future definitive cRCT of the New Start intervention, including assessment of recruitment methods, intervention delivery, stroke survivor outcome measures (completeness, follow-up rates, potential for effectiveness, estimation of the intracluster correlation coefficients (ICCs)) and safety. Carer outcomes including burden were also evaluated as part of the feasibility trial; these are provided in the Supplementary data for reference (Table O and Table P) and are fully reported elsewhere [13].

## Methods

The methods are described in full in the previously published trial protocol paper [14], with methods relevant to this paper summarised below.

### Study design

A feasibility cluster randomised trial was undertaken in English and Welsh stroke services. Stroke services (clusters) were randomised on a 1:1 basis to implement New Start or continue with usual care only. New Start was delivered by trained facilitators and offered to all stroke survivors within services allocated to the intervention. Stroke survivors were invited to participate in the trial (i.e. to complete outcome questionnaire booklets) by post. Outcome measures were collected via post at 3, 6 and 9 months after recruitment. The time-point of nine months was chosen as a pragmatic and cost-efficient final time point for this feasibility trial to allow time for attrition and impact on outcomes. The trial opened for recruitment on 26th January 2017 and closed on 30th September 2017. Follow-up was completed on 28th July 2018.

### Site selection

Stroke services were eligible if they encompassed primary and secondary care over a defined geographical area within the UK and included a stroke unit.

Additional eligibility criteria included agreement to implement a robust mechanism to identify all stroke survivors at 4 to 6 months post-stroke and the facilities and capacity to deliver New Start (i.e. staff available to undertake training and provide face-to-face contact with community-based stroke survivors at least 6 months post-stroke). Stroke services were excluded if they had previously participated in research contributing to the development of New Start or if they were currently/intending to implement a service comparable with New Start (e.g. a self-management-focussed approach) within the study duration.

**Fig. 1:**
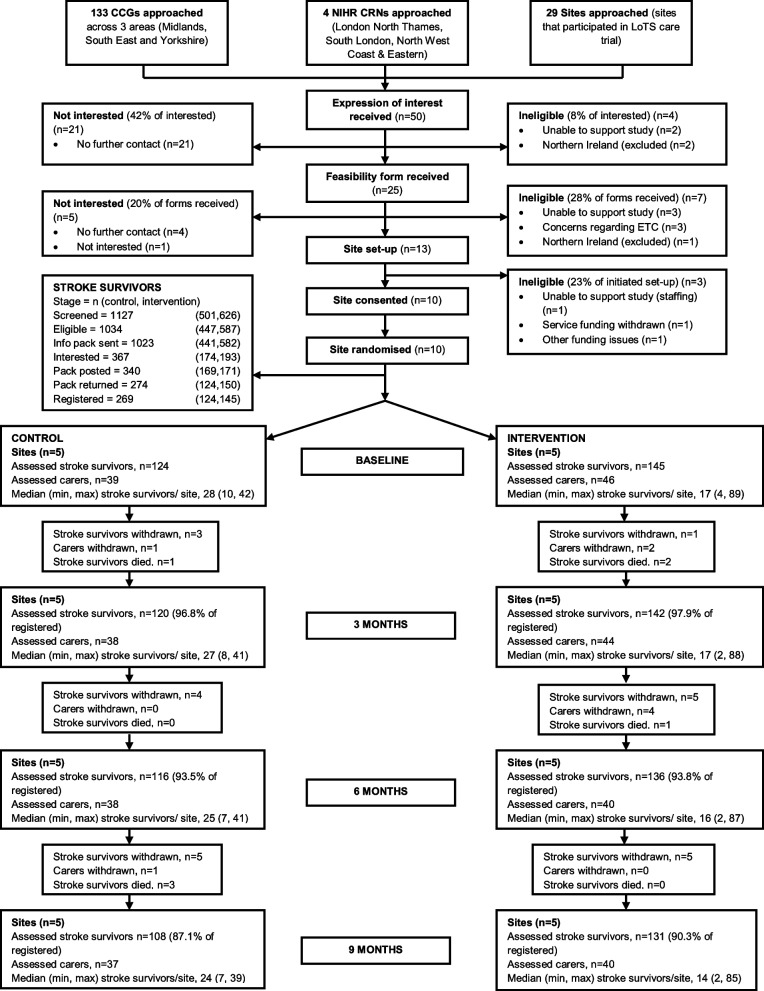
CONSORT diagram showing flow of stroke services and participants throughout trial (where “withdrawn” indicates participant withdrawal from participating in trial data collection)

Several approaches were used to identify stroke services for participation (see CONSORT diagram, Fig. 1). Clinical commissioning groups (CCGs) covering three geographical areas were contacted (*n* = 133) as well as NIHR Clinical Research Networks (CRNs) covering four areas. In addition, services that had participated in our previous (unrelated) stroke trial (LoTS care [15]) were also approached.

### Randomisation

Stroke services (clusters) were randomised sequentially, in the order recruited to the trial, using a computer-generated minimisation programme incorporating a random element, conducted centrally by the Leeds Institute of Clinical Trials Research Clinical Trials Research Unit. Minimisation factors were as follows:

1. The number of stroke survivors seen by community teams per annum (above and below the median across all recruited services)
2. Whether recruitment and intervention were delivered at separate trusts (yes/no)

Where possible, recruiting teams were not informed of the randomisation result, in order to minimise selection bias during recruitment. In one intervention site, where blinding was not feasible due to the nature of the service configuration, the recruiting team were informed of their site’s allocation.

### Intervention

#### New Start

New Start is a programme of facilitated self-management designed to be delivered at 6 months post-stroke. New Start was the result of an extensive programme of intervention development based on the Medical Research Council (MRC) framework [16] including the following: multiple literature reviews; in-depth qualitative interviews with stroke survivors and carers to identify needs, barriers and facilitators; survey of services and focus groups with staff; intervention development supported by an expert reference group and Programme Management Group initially using intervention mapping and subsequently focused on consolidated performance objectives; and, pilot work in three sites. Further details are reported elsewhere [13, 17]. The key components of New Start are listed in Table 1, and details, including a logic model of the developed intervention, are provided in Supplementary material 1. Stroke services randomised to the intervention identified New Start facilitator(s) who were trained in the intervention. Training comprised attending a structured training course involving face-to-face training supported by additional written materials. Facilitators learnt relevant theory about the self-management approach and communication skills, as well as specific details about the intervention and how to deliver it to stroke survivors.

**Table 1:** Key components of New Start^a^

• Needs assessment delivered through a face-to-face review at approximately 6 months post-stroke
• Supported self-management care strategy
• Materials to support needs assessment, self-management, goal setting and action planning, as well as the provision of useable information (the “priming tool” and “New Start guide”)
• Structured training programme for staff (face-to-face modules, supported by training worksheets and video content, as well as online learning resources)

^a^Further details regarding New Start are provided in the supplementary data

Approximately 16 weeks after completing the initial training course (to allow time for the intervention to become integrated into service provision), New Start facilitators were assessed for competency in intervention delivery, through review of patient activity records, reflective reports and telephone interviews. They were assessed using structured guides on their knowledge and understanding of the New Start care strategy, including key purposes, key features, key activities and ways it differed from their usual ways of working, and had to demonstrate an ability to apply their knowledge in practice. It was the intention to also observe facilitators delivering New Start as part of the competency assessment, but due to practical difficulties, this was only possible in three services.

New Start was to be offered to all stroke survivors within stroke services allocated to the intervention. Stroke survivors due a review (around 6 months) were to be identified and approached following local processes in each service. Each service developed their own procedures for approaching stroke survivors (e.g. appointment letter, telephone call). Reasons for not being offered or provided New Start were documented, to inform procedures for a future definitive trial. On the first visit at approximately 6 months post-stroke (aided by a priming needs assessment questionnaire), the trained facilitator elicited the stroke survivor’s needs and mapped their social networks. An open process sought to enable stroke survivors to address their identified needs and included components of problem-solving and self-management supported by New Start materials [17].

#### Usual care

Stroke services randomised to usual care continued to deliver care as determined by local policy and practices. Usual care patient activity records were completed by clinical staff to record any stroke care that was offered to patients between 6 and 12 months post-stroke, and participating services were asked to complete site surveys at regular intervals throughout the trial to indicate if there had been any changes to usual service delivery.

### Recruitment

#### Stroke survivors

Trial screening and recruitment of stroke survivors were undertaken by research staff, blind to treatment allocation (where possible) and independent of site staff delivering any interventions to participants (in either arm). In order to minimise treatment bias, New Start facilitators and usual care staff were not informed which of their patients were participating in the trial. Eligible stroke survivors were asked to consent to outcome assessment completion and permit access to their electronic healthcare records. They were informed that their stroke service was taking part in a trial of a new approach to addressing the needs of stroke survivors and their carers after a stroke (compared with usual care).

Stroke survivors were eligible for inclusion in the study if they met the criteria as follows:

1. Were between 4 and 6 months since confirmed primary diagnosis of new stroke.
2. Resided in the community (i.e. not in a nursing or residential care home).
3. Lived within the defined population covered by the stroke service.
4. Provided informed consent or consultee declaration.
5. Returned a completed baseline questionnaire.

No exclusion criteria were applied.

Screening and approaching stroke survivors for trial inclusion commenced at all stroke services approximately 12 weeks after services had been randomised to allow for New Start intervention training. A consecutive sample of stroke survivors was identified by clinical or research staff (providing that governance procedures allowed the research staff to access the clinical records) and screened for eligibility; those eligible were initially approached via a trial invitation letter. Research staff had the option to follow up any nonresponders via telephone. Interested stroke survivors were provided with a baseline questionnaire pack and consent form, by their preferred method (face to face or by post). Carer involvement in the trial was optional. All carers identified by the stroke survivor as the main informal caregiver (providing the stroke survivor with support a minimum of once per week) and who provided consent (implied via return of completed baseline questionnaire) were eligible for study inclusion.

### Sample size

As this was a feasibility study, formal power calculations were not considered appropriate. The aim was to recruit 200 stroke survivors from 10 sites over a period of approximately 6 months on the expectation that sites would be able to recruit three to four stroke survivors per month. This was deemed to be a reasonable expectation based on usual throughput of stroke services (approximately 400 per annum for a medium-sized service), and proportion of stroke survivors likely to survive to a year (60% [7]) and not be referred to a care home (80%).

### Data collection

Data were collected at the level of the service and from individual consenting participants at baseline, 3, 6 and 9 months post-recruitment.

#### Stroke survivor outcomes

Baseline assessments were administered by research staff in person or by post (according to stroke survivor preference), and follow-ups were by post at 3, 6 and 9 months post-recruitment. Follow-ups were supported by postal, telephone and text reminders, if questionnaires were not returned within 2 weeks. Proxy completion of questionnaires was permitted. If outcome measures were not returned by post, telephone interviews were conducted to maximise data collection.

A summary of the assessment schedule is provided (see Supplementary file, Table A).

The outcomes collected included measures of the following: disability/activities and participation (World Health Organization Disability Assessment Schedule 2.0, 36-item version (WHODAS 2.0)) [18–20], mental well-being (Warwick-Edinburgh Mental Well-being Scale (WEMWBS)) [21–24], patient activation (13-item Short-Form Patient Activation Measure Survey (PAM survey)) [25, 26] and longer-term unmet needs (Longer-term Unmet Needs after Stroke tool (LUNS)) [27]. The questionnaires also included relevant questions on informal and formal support received, adapted from the Millennium Survey of Poverty and Social Exclusion [28] and the GP Patient Survey [29]. A resource use questionnaire including questions on health, social care and voluntary- or third-sector service use and costs was also included to inform the health economic evaluation.

A study within a trial (SWAT) was conducted to determine the most acceptable questionnaire format to maximise follow-up rates for a future definitive trial. Stroke survivors were randomised (1:1) by the CTRU, prior to the 6-month follow-up time point, to receive one of two alternative questionnaire formats at the 6- and 9-month follow-up time points: (1) a single comprehensive booklet containing all measures or (2) two shorter booklets (one containing the outcome measures, the other containing the resource use questionnaire). The results of this are reported elsewhere [30].

#### Stroke service

Participating services were requested to complete a site survey documenting current service provision when expressing interest in the study and then at pre-randomisation (baseline), pre-recruitment and 3 monthly during recruitment and follow-up. This survey captured usual care and assessment and changes in stroke service provision during the trial (aside from New Start).

#### New Start data

Training sessions were observed and fully documented (video recordings and field notes). All contacts between the research team and facilitators regarding the implementation of the intervention were recorded (considered as implementation enhancement activities). These data were interpreted alongside the parallel process evaluation [17], providing a comprehensive evaluation of training and implementation processes. Implementation of New Start was subject to observations at each site in homes and/or clinics throughout the trial (by the process evaluation team) and monitored through regular collection of activity records, used by facilitators to record intervention delivery. These records enabled audit of the number of stroke survivors in receipt of New Start and assessment of adherence to, and fidelity of, the intervention delivery.

#### Usual care data

The site survey captured details of usual care at each participating site. Stroke services (intervention and control) were asked to record their procedures for offering 6-month reviews including means of identification and methods of contact (phone/mail). Stroke service clinical staff in all participating sites were asked to keep a usual care activity record for each stroke survivor they offered/provided a service to 6 months after their stroke. They could record whether or not the stroke survivor could be contacted and whether or not they agreed to having a review as well as details of the input received where applicable.

#### Safety data

Data on related and unexpected serious adverse events were collected. Data on hospitalisations were collected, and death of a stroke survivor/carer and institutionalisation were also recorded as expected events. Any relevant safety events were reported to the research ethics committee, to the steering committee and sponsor.

### Statistical analysis

Stroke survivor outcome data were analysed on the intention-to-treat population, defined as all participants recruited, regardless of noncompliance with the protocol or withdrawal from the study. The analyses were primarily focussed on descriptive statistics and confidence interval (CI) estimation rather than on formal hypothesis testing, with the exception of the pre-planned preliminary estimate of effectiveness.

Two-stage cluster-level analysis was used to account for the small number of clusters and small sample size per cluster [31]. Initially, point estimates based on cluster-level summaries in each arm were calculated. In the second stage, unpaired *t*-tests of the cluster-level summaries were performed at the 5%, 33% and 49% significance level. Corresponding 95%, 67% and 51% CIs for the intervention effect (mean difference) were also estimated, as it is recommended that a range of CI should be considered when reporting pilot or feasibility studies [32]. ICC estimates and 95% CI for ICC overall and by arm were also calculated to aid sample size estimation for a future definitive trial.

All data were analysed in accordance with a pre-specified analysis plan that was finalised before unblinded outcome data were available.

### Criteria for continuation to the definitive RCT

Criteria for progression to a definitive RCT were predefined and based on recruitment, follow-up and intervention implementation and delivery. A traffic-light system of green (go), amber (review) and red (stop) was applied (see Table 2).

**Table 2:** Criteria for continuation to the definitive RCT

Criteria	Green	Amber	Red
**Average recruitment of participants per site over 6 months**	≥ 20 (range 12–30)	< 20 but ≥ 10	< 10
**Return of follow-up questionnaires (at 9 months)**	≥ 75%	< 75% but ≥ 6 0%	< 60%
**Intervention training**	At least 2 members of staff from each stroke service attending training days and assessed as competent		
**Intervention delivery (% of recruited stroke survivors offered at least one session of the intervention)**	≥ 75%	< 75% but ≥ 50%	< 50%
**Intervention implementation (% of stroke services deemed competent and went on to deliver the intervention to participants)**	≥ 80% (i.e. 4 services)	60% (i.e. 3 services)	< 60% (i.e. 2 or less services)

## Results

### Stroke service recruitment

Ten stroke services were recruited from the fifty that responded to the initial contact with an expression of interest (see Fig. 1). Five services were randomised to each arm, with minimisation characteristics balanced across arms.

### Stroke survivor recruitment

Figure 1 shows the flow of participants through the trial. Two-hundred and sixty-nine stroke survivors were registered to participate in the study (23.9% of screened, 26.0% of eligible). Control sites recruited *n* = 124 stroke survivors compared with *n* = 145 in the intervention sites. The number of stroke survivors recruited per site ranged from 4 to 89 (New Start, 4–89; usual care, 10–42; see Supplementary file, Table B), with the mean number of stroke survivor participants per site being slightly higher in the intervention arm (New Start, 29; usual care, 25). Registered stroke survivors had similar characteristics to the whole screened population (see Supplementary file, Table C).

### Participant retention

Overall retention of stroke survivors during the study period was high, with 239 out of 269 (88.8%) participants being available for follow-up at 9 months (see Fig. 1). The proportion of available stroke survivors varied considerably across services (50.0–95.5%; see Supplementary file, Table D). Losses to follow-up were due to deaths (*n* = 7) or withdrawals from the study (*n* = 23) (see Fig. 1).

### Baseline data

Table 3 reports some key baseline characteristics and measures for recruited stroke survivors (see Supplementary file, Table E for additional baseline data). Baseline characteristics were broadly similar across the two arms; however, there were some notable differences in baseline measures. Participants in the New Start arm had marginally less disability as reported by WHODAS, better mental well-being as reported by WEMWBS and higher levels of patient activation as reported by PAM scores than participants in the usual care arm. Participants in the New Start arm were more likely to respond “a lot” to all of the included social questions from the Millennium Survey of Poverty and Social Exclusion, which ask how much support a person would currently receive in various situations (see Supplementary file, Table E). Conversely, a higher proportion of New Start participants reported five or more unmet needs [60/145 (41.4%) vs. 41/124 (33.1%)]. Additionally, there was substantial missing data for language ability, mRS at discharge and NIHSS at admission; because of information governance issues, mRS and NIHSS could not be supplied by one site that was a large recruiter.

**Table 3:** Baseline demographic characteristics and measures of stroke survivors by treatment arm

	**New Start (*n* = 145)**	**Usual care (*n* = 124)**	**Total (*n* = 269)**
**Age**, years (sd)	72 (11.0)	73 (12.0)	73 (11.0)
**Gender**, *n* female (%)	64 (44.1)	54 (43.5%)	118 (43.9)
Missing	0 (0.0)	1 (0.8)	1 (0.4)
**Ethnicity**, *n* (%)			
White	115 (79.3)	78 (62.9)	193 (71.7)
Other	2 (1.4)	6 (4.8)	8 (3.0)
Not stated/missing	28 (19.3)	40 (32.3)	68 (25.3)
**Living arrangement**, *n* (%)			
Living alone	40 (27.6)	34 (27.4)	74 (27.5)
Living with another person	100 (69.0)	74 (59.7)	174 (64.7)
Missing	5 (3.4)	16 (12.9)	21 (7.8)
**Education level**, *n* (%)			
None	2 (1.4)	2 (1.6)	4 (1.5)
Primary school	5 (3.4)	7 (5.6)	12 (4.5)
Secondary school	74 (51.0)	55 (44.4)	129 (48.0)
Further/higher education	61 (42.1)	56 (45.2)	117 (43.5)
Missing	3 (2.1)	4 (3.2)	7 (2.6)
**Time since stroke**, months (sd)	5.4 (0.71)	5.3 (0.83)	5.4 (0.77)
*Missing, n (%)*	*1 (0.7)*	*1 (0.8)*	*2 (0.7)*
**Level of language ability after stroke**, *n* (%)			
Normal	47 (32.4)	40 (32.3)	87 (32.3)
Dysphasia/dysarthria	8 (5.5)	1 (0.8)	9 (3.3)
Not known/missing	90 (62.1)	83 (66.9)	173 (64.3)
**Length of hospital admission,** days (sd)	11 (18)	15 (24)	13 (21)
*Missing, n (%)*	*4 (2.8)*	*9 (7.3)*	*13 (4.8)*
**WHODAS**^a^			
**Simple score,** points (sd)	23.7 (18.1)	26.2 (20.8)	24.9 (19.4)
*Missing, n (%)*	*28 (19.3)*	*20 (16.1)*	*48 (17.8)*
**Complex score**, points (sd)	25.6 (19.3)	26.9 (24.3)	26.2 (21.7)
*Missing, n (%)*	*68 (46.9)*	*56 (45.2)*	*124 (46.1)*
**WEMWBS score**^b^, points (sd)	47.5 (11.6)	46.6 (12.6)	47.1 (12.1)
*Missing, n (%)*	*0 (0.0)*	*6 (4.8)*	*6 (2.2)*
**PAM score**^c^, points (sd)	58.6 (17.7)	56.7 (16.9)	57.7 (17.4)
*Missing, n (%)*	*2 (1.4)*	*3 (2.4)*	*5 (1.9)*
**PAM level (categorized PAM score)**, *n* (%)			
(< = 47.0) not believing activation important	36 (24.8)	38 (30.6)	74 (27.5)
(47.1–55.1) a lack of knowledge and confidence to take action	45 (31.0)	32 (25.8)	77 (28.6)
(55.2–67.0) beginning to take action	29 (20.0)	30 (24.2)	59 (21.9)
(> = 67.1) taking action	33 (22.8)	21 (16.9)	54 (20.1)
Missing	2 (1.4)	3 (2.4)	5 (1.9)
**LUNS (number of long-term unmet needs)**, *n* (%)			
0	16 (11.0)	19 (15.3)	35 (13.0)
1–4	69 (47.6)	62 (50.0)	131 (48.7)
≥ 5	60 (41.4)	41 (33.1)	101 (37.5)
Missing	0 (0.0)	2 (1.6)	2 (0.7)

Values reported are mean (sd) unless otherwise stated

^a^Higher score indicates higher level of disability

^b^higher score indicates better state of wellbeing

^c^higher score indicates higher level of activation

### New Start delivery

Fifteen facilitators were identified to deliver New Start across five sites (range 2–4 facilitators per site). Most facilitators attended the initial training session (*n* = 14) and follow-up session (*n* = 13). Face-to-face training was provided at a later date to one facilitator who had not attended the initial session. All facilitators were assessed as competent in intervention delivery.

In keeping with the cluster trial design, all stroke survivors at intervention sites were offered the intervention, not all of whom consented to outcome data collection. Across all intervention sites, at least 541 stroke survivors were contacted regarding New Start, and 69.7% (*n* = 377) went on to receive at least one session. Among trial participants, 138 out of 145 (95.2%) stroke survivors were contacted regarding New Start, and 86 of these (59.3%) went on to receive at least one New Start session. These participants attended an average of 1.14 meetings, each lasting approximately 1 h (see Table 4).

**Table 4:** Delivery of New Start intervention by site

Site	Recruited stroke survivors (*n*)	Stroke survivors offered intervention (*n*, % of recruited)	Stroke survivors received at least one New Start session (*n*, % of recruited)	Total number of New Start sessions	Average number of sessions per stroke survivor	Average duration of session (min)
1	13	11 (84.6)	7 (53.8)	13	1.86	80.4
2	4	4 (100)	3 (75.0)	3	1	51.7
3	89	87 (97.8)	65 (73.0)	71	1.09	57.0
4	22	22 (100)	9 (40.9)	9	1	41.7
5	17	14 (82.4)	2 (11.8)	2	1	60.0
Total	145	138 (95.2)	86 (59.3)	98	1.14	58.6

Baseline characteristics were compared between trial participants who received at least one New Start session and those who did not receive any New Start sessions (either because they were not offered it, they actively declined it or they did not respond to the initial invitation) (see Supplementary file, Table F). Those who did not receive a New Start session were more likely to have received further/higher education (44.1% (26/59) vs. 40.7% (35/86)) and to have reported lower levels of unmet needs (33.9% (20/59) vs. 46.5% (40/86) reporting ≥ 5 unmet needs). Those receiving a New Start session had, on average, a longer stay in hospital as a result of their stroke (13 vs. 8 days).

### Usual care

Four of the five usual care sites offered a 6-month review as part of the usual stroke care pathway, compared with all five intervention sites. Across usual care sites, 86.1% (93/108) of stroke survivors who were offered stroke care between 6 and 12 months post-stroke were seen or spoken to; the majority (87.1%) of these had one contact, with an average duration of contact of 54 min (see Supplementary file, Table G). Three out of five intervention sites provided stroke care additional to New Start between 6 and 12 months post-stroke, and 16.6% (24/145) of trial participants received contact not related to New Start. The number of contacts per stroke survivor varied between sites, and average duration of contacts was 44 min (see Supplementary file, Table G). The majority of intervention trial participants (*n* = 105) were not offered any additional stroke care between 6 and 12 months post-stroke.

### Unblinding

There were 14 reported occasions of New Start facilitator unblinding to their patients’ research participation (usually through patients informing the facilitators of trial engagement), occurring across three sites and 13 patients (see Supplementary file, Table H, for further details).

### Assessment of outcome measures

#### Completion rates

At each time point for all questionnaires, completion rates in the New Start arm were slightly higher than those in the usual care arm.

There was a higher proportion of completed questionnaires at 9 months for the 14-item WEMWBS than for the 36-item WHODAS (77.8% v 20.1% of available participants) (see Supplementary file, Table I). However, the flexibility of the WHODAS simple score meant that, at 9 months, the proportion of partially completed questionnaires that could not be scored was higher for the WEMWBS than for the WHODAS with simple scoring (44.0% vs. 20.0%). This was not the case for the more rigid complex scoring of the WHODAS, for which 50.0% of partially completed questionnaires could not be scored because of missing items.

Of the partially completed PAM questionnaires at 6 months, 47.6% (20/42) could not be scored because of missing items.

#### Statistical outcomes

Stroke survivor questionnaire outcomes are reported at both participant level (see Supplementary file, Table J) and cluster level. Significance testing to explore preliminary estimates of effectiveness is reported on the cluster-level point estimates of the WHODAS and WEMWBS scores (see Table 5). At the 5% significance level, there was no evidence of a difference between treatment arms in WHODAS and WEMWBS scores. At 6 months, there was an indication of a lower level of disability as measured by the WHODAS (simple score) in the New Start arm than in the usual care arm (difference 3.14, 67% *CI* 0.76 to 5.51, 51% *CI* 1.48 to 4.79) at the 33% and 49% significance levels; however, this was not seen at 9 months.

**Table 5:** *T*-test results for WHODAS and WEMWBS cluster-level scores

	**Time point**	**Cluster point estimates**				**Mean difference (*CI*: 95%, 67%, 51%)**	***T*-test *p*-value**^**a**^ **Significant at 5%, 33%, 49% level**
		**New start**		**Usual care**			
		**n/N**^**b**^	**Mean (SD)**	**n/N**	**Mean (SD)**		
**WHODAS simple** *(score 0–100; higher score* = *higher level of disability)*	Baseline	4/5	26.2 (4.34)	5/5	23.9 (5.81)	-2.3	0.53
						(-10.60, 6.01)	No
						(-5.97, 1.38)	No
						(-4.85, 0.26)	No
	6 months	4/5	21.12 (2.93)	5/5	24.2 (3.69)	3.14	0.21
						(-2.23, 8.50)	No
						(0.76, 5.51)	Yes
						(1.48, 4.79)	Yes
	9 months	4/5	24.2 (4.71)	5/5	23.34 (4.38)	-0.87	0.52
						(-8.05, 6.30)	No
						(-4.05, 2.30)	No
						(-3.08, 1.34)	No
**WHODAS complex** *(score 0–100; higher score* = *higher level of disability)*	Baseline	4/5	28.0 (5.34)	5/5	24.7 (7.72)	−3.26	0.49
						(-14.05, 7.53)	No
						(-8.04, 1.51)	No
						(-6.59, 0.06)	No
	6 months	4/5	23.9 (4.56)	5/5	26.0 (6.89)	2.07	0.62
						(-7.46, 11.59)	No
						(-2.15, 6.29)	No
						(-0.87, 5.00)	No
	9 months	4/5	26.2 (6.22)	5/5	26.0 (5.99)	-0.16	0.97
						(-9.82, 9.50)	No
						(-4.44, 4.11)	No
						(-3.14, 2.82)	No
**WEMWBS** *(score 14–70; higher score* = *better state of mental well-being)*	Baseline	4/5	46.9 (2.06)	5/5	47.2 (1.55)	0.29	0.82
						(-2.54, 3.12)	No
						(-0.96, 1.54)	No
						(-0.58, 1.16)	No
	6 months	4/5	47.2 (1.80)	5/5	44.4 (5.70)	-2.87	0.37
						(-9.96, 4.21)	No
						(-6.01, 0.26)	No
						(-5.06, − 0.69)	Yes
	9 months	4/5	45.8 (2.71)	5/5	47.2 (2.80)	1.42	0.47
						(-2.96, 5.80)	No
						(-0.52, 3.36)	No
						(0.07, 2.77)	Yes

^a^Unpaired t-tests were performed at three different significance levels (5%, 33% and 49%) on cluster-level questionnaire data at each time point, producing corresponding confidence intervals (at 95%, 67% and 51%). These results are not adjusted for baseline scores. For data completeness, see Table I in Supplementary data

^b^Only four of the five sites in the intervention arm were analysed at cluster level. This is due to one site containing a very small number of stroke survivors, and thus not providing enough information about the cluster for accurate analysis

The bubble plots in Fig. 2 (see Supplementary file) summarise, by arm, the number of participants with a change in PAM level between timepoints. Between baseline and 6 months, 26.5% (30/113) of stroke survivors in the New Start arm with complete data reported an increase in their PAM level, compared with only 20% (17/85) of usual care participants. This pattern was also seen between 3 and 6 months [New Start, 28.8% (32/111); usual care, 26.8% (22/82)] and between baseline and 3 months [New Start, 23.4% (29/124); usual care, 20.0% (20/100)]. It is important to note, however, that there was a higher proportion of missing PAM scores in the usual care arm than the New Start arm at 6 months (29.8% vs. 20.7%). The mean PAM scores in both groups (Supplementary file, Table J) did not indicate change in line with the reported 4-point minimal clinically important difference [33].

### Sample size estimation for a definitive study

The mean cluster size was 29 (*SD*, 34.18; coefficient of variation 1.18) in the New Start arm and 25 (*SD*, 12.64; coefficient of variation, 0.51) in the usual care arm. The higher level of variance observed in the New Start arm is due to one small site (site 2, *n* = 4) and one large site (site 3, *n* = 89). Omitting the small site from the calculations, as was done in the cluster-level significance tests, results in a mean cluster size in the intervention arm of 35 (*SD*, 36.02; coefficient of variation, 1.02).

A range of intracluster correlation coefficients, as well as estimates for the coefficient of within-cluster variation, were calculated for the WHODAS (simple and complex scores) and the WEMWBS questionnaires at each of the data collection timepoints. Again, the small site was omitted from the calculations. Because of the small number of clusters and participants, it was difficult to produce reliable ICC estimates. The range of ICC values produced was between 0.01 and 0.02 (see Supplementary file, Table K).

### Safety

There were no trial-related safety concerns. Seven deaths were recorded in the population during the study period: four in the usual care arm (3.2%) and three in the New Start arm (2.1%). For further details on hospitalisations and institutionalisations, see Supplementary file, Table L.

### Progression to a definitive trial

Green targets were achieved for most of the predetermined criteria for progression to a definitive trial: target stroke survivor recruitment rates were achieved, on average, across sites (24.1 per site over 6 months, see Supplementary file, Table M); target follow-up rates were achieved with 216 (80.3%) registered stroke survivors returning follow-up questionnaires at 9 months (84.1% in the intervention arm and 75.8% in the usual care arm, see Supplementary file, Table N); and targets related to intervention delivery were also achieved with all five intervention sites having at least two facilitators trained and deemed competent and going on to deliver the New Start intervention. Overall, 95.2% of registered stroke survivors were offered the intervention (see Table 4).

However, there were concerns regarding the number of stroke survivors receiving at least one session of the intervention at some sites. In particular, at sites 4 and 5, only 40.9% and 11.8% of recruited stroke survivors received at least one session of New Start, respectively (see Table 4). Uptake was largely influenced by method of invitation, location of delivery and pre-existing services. At site 5, New Start was offered as an optional extra in addition to the standard 6-month review already delivered to stroke survivors. At this site, stroke survivors received a letter inviting them to telephone and make an appointment at a hospital-based clinic to receive New Start. At site 4, the offer of a review was made by telephone, as an opt-in clinic appointment. Facilitators at this site made a clinical decision whether to deliver a standard review or New Start.

## Discussion

A cluster randomised controlled feasibility trial of New Start was successfully completed, suggesting that the trial processes employed were generally feasible. Ten stroke services were recruited to take part in the trial, and these sites recruited a total of 269 stroke survivors to complete outcome assessments. In the five intervention services, facilitators were appointed, successfully trained and went on to deliver New Start. No safety concerns were identified, and return rates of at least 75% were achieved for the outcome assessments at 9 months, with retention of 88.8% of participants for the study period.

### Stroke service recruitment

A number of approaches were used to identify stroke services for participation, including contacting CCGs, NIHR CRNs and twenty-nine services that had participated in our previous (unrelated) trial [15]. Although some CCGs expressed interest, the translation from interest to recruitment was low, primarily because the initial expression of interest was removed from the clinical setting. A number of sites in the CRNs expressed interest, but did not appreciate the requirement for clinical engagement in intervention delivery. The most successful approach was to sites who had engaged in our previous work.

The ten recruited stroke services reflected a wide geographical and cultural spread from across England and Wales. Of the ten stroke services, nine provided 6-month reviews as part of their usual service prior to the study. The level of service provision up to 6 months post-stroke varied considerably between the services, demonstrating even in this small sample the wide variety of support offered to people post-stroke.

### Stroke survivor recruitment

Recruiting participants to longer-term stroke studies has previously been challenging, as there is no central register of stroke survivors and, once provision of services is ended, which might be soon after stroke for some, there are limited opportunities to identify and engage with this population. We believe that this study is one of the first to establish procedures to enable recruitment of a cohort of post-discharge stroke survivors. However, it is important to note that in this feasibility trial, our procedures and information governance protocols did not always enable collection of data, for example on language ability, if recruiting staff did not have or were not allowed access to in-patient medical records.

The overall recruitment rate (24.1 participants per site, prorated to a 6-month period) fulfilled the green recruitment criterion for progression to a definitive trial. On average, sites recruited 4.0 stroke survivors per month, which compares favourably with previous stroke rehabilitation RCTs [34]. However, there was considerable variation in the numbers screened and recruited between stroke services. Services were randomised with minimisation characteristics, including number of stroke survivors seen by community teams per annum, balanced between arms. Despite this, however, one intervention site had considerably higher recruitment levels than the other sites. Other sites had particularly low recruitment levels, some of which could be explained (rural population with lower caseload), others of which could not (urban area with potential for larger throughput), despite exploring potential factors (e.g. number of competing trials, socioeconomic aspects of the population).

The majority of stroke survivors screened for participation were eligible (91.7%), and uptake was reasonable at 26.0%, comparable with other longer-term stroke trials [31]. Registered stroke survivors had similar characteristics to the whole screened population, and the majority of the participants had no further education since leaving school (54%), which is similar to previous study cohorts [35]. This suggests our approach recruited a population representative of stroke survivors that would support generalisability of results in a future trial. However, incomplete data on language ability after stroke reduces our certainty about this.

### Intervention delivery

Facilitators were successfully appointed and trained to deliver New Start across all intervention services. In order to minimise treatment bias, clinical staff (in both arms) were not informed which of their patients were participating in the trial. Due to the necessary separation of recruitment and clinical processes, we had some concern that there would be a mismatch between the stroke survivors offered the intervention and those invited into the trial; fortunately, this was ill-founded, with the majority of trial participants (95.2%) being offered the intervention. However, only 59.3% of trial participants actually went on to receive at least one New Start session. To be inclusive and because of the dearth of any contrary evidence, we sought to offer the intervention to all stroke survivors. Perhaps inevitably, this meant that the population was heterogeneous; some people felt that they no longer required input from stroke services. It is of note that the people who did not receive at least one New Start session (mainly due to actively declining it or not responding to the initial invitation) on average had slightly higher levels of higher education, shorter length of hospital stay and lower levels of unmet need than those who did receive a session. In addition, uptake was negatively influenced by method of invitation (opt-in by telephone vs opt-out by letter), location of delivery (clinic vs home) and the presence of competing pre-existing services. Any future evaluation of the intervention would benefit from more focused targeting and consistent approaches to optimise uptake and ensure a higher proportion of trial participants receive at least one session of the intervention.

In those participants who agreed to receive New Start, the dose received was less than had been intended (1.14 sessions on average per stroke survivor compared with at least two sessions (initial and follow-up) as intended). Delivery of the intervention was suboptimal in some sites and will need addressing prior to a definitive trial. The in-depth process evaluation report provides further insights into the delivery of New Start [13].

### Outcome assessment

Follow-up was largely successful, with rates at 9 months (80.3%) fulfilling the green follow-up criterion for progression to a definitive trial. Increase in withdrawal at the 9-month assessment point and participants indicating that they had nothing new to report and therefore had not returned the assessment booklet suggest that the follow-up assessments were too close. Of those that were returned, there was a higher proportion of completed questionnaires for the 14-item WEMWBS than for the 36-item WHODAS indicating that length of questionnaire had an influence on completion.

Although potential efficacy was seen, with an indication of a lower WHODAS simple score in the New Start arm than in the usual care arm at 6 months, it is not possible to draw conclusions from this given the feasibility design and associated small sample size.

## Conclusions

This trial has demonstrated that it is feasible to recruit stroke services and stroke survivors. Postal recruitment and outcome data collection have proven to be acceptable to longer-term stroke survivors, with few withdrawals and high follow-up rates. However, New Start requires further refinement and for the target population to be more clearly specified, for example through a prior assessment of unmet need, prior to evaluation in a large-scale trial.

## Supplementary Information

**Additional file 1:** **Fig. 2.** Change in Patient Activation Measure (PAM) category between Baseline, 3 and 6 month time points. **Table A.** Summary and timing of stroke survivor assessments. **Table B.** Stroke survivor screening flow and recruitment figures, by stroke service. **Table C.** Demographic characteristics of screened and registered participants. **Table D.** Stroke survivor follow-up availability due to withdrawals and deaths, by stroke service. **Table E.** Additional baseline characteristics and measures of stroke survivors by treatment arm. **Table F.** Baseline characteristics of participants in intervention sites split by intervention receipt. **Table G.** Description of usual care by site. **Table H.** Facilitator unblinding by site. **Table I.** Questionnaire completeness at all time points. **Table J.** Summary statistics of all outcome measures at various time points – patient-level. **Table K.** ICC estimates for patient reported outcomes. **Table L.** Hospitalisation and institutionalisation reported by stroke survivors by arm. **Table M.** Recruitment progression criteria. **Table N.** Follow up progression criteria (follow-up at 9 months). **Table O.** Baseline demographic characteristics and measures of carers by treatment arm. **Table P.** Caregiver Burden Scale (CBS) questionnaire scores for all time points.

**Additional file 2:** **Appendix 1.** Full list of LoTS2Care trial objectives.

## Data Availability

Any data requests should be sent to the corresponding author and would be subject to review by a subgroup of the trial team, which will include the data guarantor, Professor Farrin. All data-sharing activities would require a data-sharing agreement.

## Abbreviations

ADL: Activities of daily living
CCG: Clinical commissioning group
CI: Confidence interval
cRCT: Cluster randomised controlled trial
CRN: Clinical Research Network
ICC: Intracluster correlation coefficient
LUNS: Longer-term Unmet Needs after Stroke
NIHR: National Institute for Health Research
PAM: Patient activation measure
QoL: Quality of life
WEMWBS: Warwick-Edinburgh Mental Well-being Scale
WHODAS: World Health Organization Disability Assessment Schedule
