# Supplementary material for: An intervention to support stroke survivors and their carers in the longer term: results of a cluster randomised controlled feasibility trial (LoTS2Care)
Source: Pilot Feasibility Stud. 2023 Mar 15;9:40. doi: 10.1186/s40814-023-01258-6 (PMC10015731; doi:10.1186/s40814-023-01258-6)
Supplement: Supplementary file 1 — Additional file 1: Fig. 2. Change in Patient Activation Measure (PAM) category between Baseline, 3 and 6 month time points. Table A. Summary and timing of stroke survivor assessments. Table B. Stroke survivor screening flow and recruitment figures, by stroke service. Table C. Demographic characteristics of screened and registered participants. Table D. Stroke survivor follow-up availability due to withdrawals and deaths, by stroke service. Table E. Additional baseline characteristics and measures of stroke survivors by treatment arm. Table F. Baseline characteristics of participants in intervention sites split by intervention receipt. Table G. Description of usual care by site. Table H. Facilitator unblinding by site. Table I. Questionnaire completeness at all time points. Table J. Summary statistics of all outcome measures at various time points – patient-level. Table K. ICC estimates for patient reported outcomes. Table L. Hospitalisation and institutionalisation reported by stroke survivors by arm. Table M. Recruitment progression criteria. Table N. Follow up progression criteria (follow-up at 9 months). Table O. Baseline demographic characteristics and measures of carers by treatment arm. Table P. Caregiver Burden Scale (CBS) questionnaire scores for all time points. [file 40814_2023_1258_MOESM1_ESM.doc]

**LoTS2Care feasibility cRCT results paper - Supplementary Data**

**Table A**: Summary and timing of stroke survivor assessments

| **Assessment** | **Screening** | **Baseline** | **Time-point (post registration)** | | |
| --- | --- | --- | --- | --- | --- |
|  |  |  | **3**  **months** | **6**  **months** | **9**  **months** |
| Screening | X |  |  |  |  |
| Eligibility |  | X |  |  |  |
| Informed Consent /  Consultee declaration |  | X |  |  |  |
| Baseline |  | X |  |  |  |
| Registration |  | X |  |  |  |
| Demographic Details |  | X |  |  |  |
| WHODAS 2.0 |  | X |  | X | X |
| WEMWBS |  | X |  | X | X |
| PAM® Survey |  | X | X | X |  |
| LUNS |  | X |  |  | X |
| GP Patient Survey (2 questions) |  | X | X | X |  |
| Social Questions |  | X | X | X |  |
| Adverse Events |  |  | X | X | X |
| Hospital Admissions |  |  | X | X | X |
| Health and social care resource use |  | X | X | X | X |

LUNS: Longer-term Unmet Needs after Stroke tool; PAM: Patient Activation Measure; WEMWBS: Warwick-Edinburgh Mental Well-being Scale; WHODAS: World Health Organization Disability Assessment Schedule

**Table B: Stroke survivor screening flow and recruitment figures, by stroke service**

| **Site** | **Screened N** | **Eligible (of screened) N(%)** | **Info pack sent (of eligible) N(%)** | **Interested in taking part (of pack sent) N(%)** | **Baseline questionnaire posted (of interested) N(%)** | **Baseline questionnaire returned (of posted) N(%)** | **Registered (of pack returned) N(%)** | **Registered (of eligible) (%)** |
| --- | --- | --- | --- | --- | --- | --- | --- | --- |
| ***New Start sites*** | | |  |  |  |  |  |  |
| 1 | 39 | 38 (97.4) | 37 (97.4) | 16 (43.2) | 16 (100.0) | 13 (81.3) | 13 (100.0) | (34.2%) |
| 2 | 29 | 27 (93.1) | 27 (100.0) | 4 (14.8) | 4 (100.0) | 4 (100.0) | 4 (100.0) | (14.8%) |
| 3 | 350 | 347 (99.1) | 345 (99.4) | 121 (35.1) | 110 (90.9) | 93 (78.2) | 89 (95.7) | (25.6%) |
| 4 | 106 | 94 (88.7) | 92 (97.9) | 28 (30.4) | 27 (96.4) | 23 (82.1) | 22 (95.7) | (23.4%) |
| 5 | 102 | 81 (79.4) | 81 (100.0) | 24 (29.6) | 16 (66.7) | 17 (70.8) | 17 (100.0) | (16.7%) |
| ***Usual Care sites*** | | |  |  |  |  |  |  |
| 6 | 183 | 168 (91.8) | 168 (100.0) | 68 (40.5) | 67 (98.5) | 43 (64.2) | 42 (97.7) | (21.0%) |
| 7 | 30 | 29 (96.7) | 29 (100.0) | 10 (34.5) | 10 (100.0) | 10 (100.0) | 10 (100.0) | (34.5%) |
| 8 | 112 | 111 (99.1) | 110 (99.1) | 36 (32.7) | 35 (97.2) | 29 (82.9) | 29 (100.0) | (26.1%) |
| 9 | 116 | 89 (76.7) | 84 (94.4) | 42 (50.0) | 39 (92.9) | 28 (66.7) | 28 (100.0) | (31.5%) |
| 10 | 60 | 50 (83.3) | 50 (100.0) | 18 (36.0) | 18 (100.0) | 15 (83.3) | 15 (100.0) | (30.0%) |
| ***Total*** | 1127 | 1034 (91.7) | 1023 (98.9) | 367 (35.9) | 340 (92.6) | 274 (75.5) | 269 (98.2) | (26.0%) |

**Table C:** Demographic characteristics of screened and registered participants

|  | ***Recruitment Screening*** | | | ***Clinical Screening**** | | | ***Registered*** | | |
| --- | --- | --- | --- | --- | --- | --- | --- | --- | --- |
|  | **New Start**  **(n = 626)** | **Usual Care**  **(n = 501)** | **Total**  **(n = 1127)** | **New Start**  **(n = 761)** | **Usual Care**  **(n = 386)** | **Total**  **(n = 1147)** | **New Start**  **(n = 145)** | **Usual Care**  **(n = 124)** | **Total**  **(n = 269)** |
| **Age**, years (s.d.) | 73.4 (12.9) | 73.9 (13.4) | 73.6 (13.1) | 73.7 (13.1) | 72.9 (14.2) | 73.5 (13.4) | 71.6 (10.9) | 72.2 (11.65) | 71.9 (11.2) |
| *Missing, n (%)* | *1 (0.2)* | *8 (1.6)* | *9 (0.8)* | *3 (0.4)* | *23 (6.0)* | *26 (2.3)* | *0 (0.0)* | *1 (0.8)* | *1 (0.4)* |
| **Gender**, n female (%) | 269 (43.0) | 231 (46.1) | 500 (44.4) | 340 (44.7) | 166 (43.0) | 506 (44.1) | 64 (44.1) | 55 (44.4) | 119 (44.2) |
| Missing | 2 (0.3) | 6 (1.2) | 8 (0.7) | 0 (0.0) | 1 (0.3) | 1 (0.1) | 0 (0.0) | 1 (0.8) | 1 (0.4) |
| **Ethnicity**, n (%) |  |  |  |  |  |  |  |  |  |
| White | 314 (50.2) | 275 (54.9) | 589 (52.3) | 696 (91.5) | 339 (87.8) | 1035 (90.2) | 115 (79.3) | 76 (61.3) | 191 (71.0) |
| Other | 19 (3.0) | 19 (3.8) | 38 (3.4) | 30 (3.9) | 28 (7.3) | 58 (5.1) | 2 (1.4) | 6 (4.8) | 8 (3.0) |
| Not stated/Missing | 293 (46.8) | 207 (41.3) | 500 (44.4) | 35 (4.6) | 19 (4.9) | 54 (4.7) | 28 (19.3) | 42 (33.9) | 70 (26.0) |
| **Length of hospital admission,** days (s.d.) | 12 (28) | 13 (41) | 13 (34) |  |  |  | 11 (17) | 14 (20) | 12 (19) |
| *Missing, n (%)* | *146 (23.3)* | *94 (18.8)* | *240 (22.3)* |  |  |  | *5 (3.4)* | *12 (9.7)* | *17 (6.3)* |
| **mRS at discharge**, n (%) |  |  |  |  |  |  |  |  |  |
| 0 | 58 (9.3) | 20 (4.0) | 78 (6.9) |  |  |  | 12 (8.3) | 7 (5.6) | 19 (7.1) |
| 1 | 77 (12.3) | 63 (12.6) | 140 (12.4) |  |  |  | 23 (15.9) | 24 (19.4) | 47 (17.5) |
| 2 | 35 (5.6) | 82 (16.4) | 117 (10.4) |  |  |  | 7 (4.8) | 25 (20.2) | 32 (11.9) |
| 3 | 32 (5.1) | 83 (16.6) | 115 (10.2) |  |  |  | 8 (5.5) | 19 (15.3) | 27 (10.0) |
| 4 | 25 (4.0) | 51 (10.2) | 76 (6.7) |  |  |  | 2 (1.4) | 15 (12.1) | 17 (6.3) |
| 5 | 12 (1.9) | 9 (1.8) | 21 (1.9) |  |  |  | 1 (0.7) | 2 (1.6) | 3 (1.1) |
| 6 | 1 (0.2) | 0 (0.0) | 1 (0.1) |  |  |  |  |  |  |
| Missing | 386 (61.7) | 193 (38.5) | 579 (51.4) |  |  |  | 92 (63.4) | 32 (25.8) | 124 (46.1) |
| **NIHSS score at admission**, mean (s.d.) | 6.1 (6.05) | 5.6 (5.79) | 5.8 (5.90) |  |  |  | 4.4 (4.55) | 4.9 (5.46) | 4.7 (5.11) |
| *Missing, n (%)* | *375 (60.0)* | *181 (36.1)* | *556 (49.3)* |  |  |  | *88 (60.7)* | *36 (29.0)* | *124 (46.1)* |

Values reported are mean (s.d.) unless otherwise stated. Summaries are based on the data from the screening database

*data reported from post-stroke contact log completed by clinical staff for every stroke survivor they attempted to contact for follow-up.

**Table D:** Stroke survivor follow-up availability due to withdrawals and deaths, by stroke service

|  | **New Start** | | | | | | **Usual Care** | | | | | |
| --- | --- | --- | --- | --- | --- | --- | --- | --- | --- | --- | --- | --- |
|  | **1**  **(n=13)** | **2**  **(n=4)** | **3**  **(n=89)** | **4**  **(n=22)** | **5**  **(n=17)** | **Total (n=145)** | **6 (n=42)** | **7 (n=10)** | **8 (n=29)** | **9 (n=28)** | **10 (n=15)** | **Total (n=124)** |
| **Available at 3m,** n (%) | 13  (100.0) | 2  (50.0) | 88  (98.9) | 22  (100.0) | 17 (100.0) | 142 (97.9) | 41 (97.6) | 9 (90.0) | 29 (100.0) | 27 (96.4) | 14 (93.3) | 120 (96.8) |
| **Available at 6m,** n (%) | 12  (92.3) | 2  (50.0) | 87  (97.8) | 19  (86.4) | 16  (94.1) | 136 (93.8) | 41 (97.6) | 8 (80.0) | 28 (96.6) | 25 (89.3) | 14 (93.3) | 116 (93.5) |
| **Available at 9m,** n (%) | 11  (84.6) | 2  (50.0) | 85  (95.5) | 19  (86.4) | 14  (82.4) | 131 (90.3) | 39 (92.9) | 8 (80.0) | 26 (89.7) | 24 (85.7) | 11 (73.3) | 108 (87.1) |

**Table E: Additional baseline characteristics and measures of stroke survivors by treatment arm**

|  | **New Start**  **(n=145)** | **Usual Care**  **(n=124)** | **Total**  **(n=269)** |
| --- | --- | --- | --- |
| **Marital status**, n (%) |  |  |  |
| Single | 7 (4.8) | 7 (5.6) | 14 (5.2) |
| Married/Living as married | 89 (61.4) | 76 (61.3) | 165 (61.3) |
| Separated/Divorced/Widowed | 46 (31.7) | 38 (30.6) | 84 (31.2) |
| Missing | 3 (2.1) | 3 (2.4) | 6 (2.2) |
| **Time between onset/awareness of stroke and hospital admission^a^,** days (s.d.) | 0.1 (0.84) | 0.5 (4.84) | 0.3 (3.34) |
| *Missing, n (%)* | *3 (2.1)* | *4 (3.2)* | *7 (2.6)* |
| **NIHSS score at admission^b^**, points (s.d.) | 4.5 (4.5) | 5.0 (5.5) | 4.8 (5.1) |
| *Missing, n (%)* | *88 (60.7)* | *38 (30.6)* | *126 (46.8)* |
| **mRS at discharge^b^**, n (%) |  |  |  |
| 0 | 11 (7.6) | 5 (4.0) | 16 (5.9) |
| 1 | 22 (15.2) | 24 (19.4) | 46 (17.1) |
| 2 | 7 (4.8) | 27 (21.8) | 34 (12.6) |
| 3 | 8 (5.5) | 20 (16.1) | 28 (10.4) |
| 4 | 1 (0.7) | 14 (11.3) | 15 (5.6) |
| 5 | 1 (0.7) | 2 (1.6) | 3 (1.1) |
| Missing | 95 (65.5) | 32 (25.8) | 127 (47.2) |
| **Social questions (how much support)**, n (%) |  |  |  |
| ***Help around house when ill*** |  |  |  |
| A lot | 96 (66.2) | 65 (52.4) | 161 (59.9) |
| Some | 32 (22.1) | 34 (27.4) | 66 (24.5) |
| Not much | 9 (6.2) | 9 (7.3) | 18 (6.7) |
| None at all | 8 (5.5) | 13 (10.5) | 21 (7.8) |
| Missing | 0 (0.0) | 3 (2.4) | 3 (1.1) |
| ***Help with heavy jobs*** |  |  |  |
| A lot | 89 (61.4) | 63 (50.8) | 152 (56.5) |
| Some | 37 (25.5) | 32 (25.8) | 69 (25.7) |
| Not much | 10 (6.9) | 12 (9.7) | 22 (8.2) |
| None at all | 9 (6.2) | 12 (9.7) | 21 (7.8) |
| Missing | 0 (0.0) | 5 (4.0) | 5 (1.9) |
| ***Advice on important changes*** |  |  |  |
| A lot | 57 (39.3) | 44 (35.5) | 101 (37.5) |
| Some | 34 (23.4) | 29 (23.4) | 63 (23.4) |
| Not much | 15 (10.3) | 12 (9.7) | 27 (10.0) |
| None at all | 20 (13.8) | 25 (20.2) | 45 (16.7) |
| Missing | 19 (13.1) | 14 (11.3) | 33 (12.3) |
| ***Problems with spouse*** |  |  |  |
| A lot | 58 (40.0) | 37 (29.8) | 95 (35.3) |
| Some | 33 (22.8) | 24 (19.4) | 57 (21.2) |
| Not much | 8 (5.5) | 9 (7.3) | 17 (6.3) |
| None at all | 18 (12.4) | 25 (20.2) | 43 (16.0) |
| Missing | 28 (19.3) | 29 (23.4) | 57 (21.2) |
| ***Feeling depressed*** |  |  |  |
| A lot | 68 (46.9) | 45 (36.3) | 113 (42.0) |
| Some | 45 (31.0) | 43 (34.7) | 88 (32.7) |
| Not much | 12 (8.3) | 18 (14.5) | 30 (11.2) |
| None at all | 13 (9.0) | 13 (10.5) | 26 (9.7) |
| Missing | 7 (4.8) | 5 (4.0) | 12 (4.5) |
| ***Help caring for someone*** |  |  |  |
| A lot | 40 (27.6) | 30 (24.2) | 70 (26.0) |
| Some | 16 (11.0) | 15 (12.1) | 31 (11.5) |
| Not much | 4 (2.8) | 6 (4.8) | 10 (3.7) |
| None at all | 24 (16.6) | 29 (23.4) | 53 (19.7) |
| Missing | 61 (42.1) | 44 (35.5) | 105 (39.0) |
| ***Need someone to look after your home when away*** |  |  |  |
| A lot | 71 (49.0) | 52 (41.9) | 123 (45.7) |
| Some | 34 (23.4) | 31 (25.0) | 65 (24.2) |
| Not much | 5 (3.4) | 12 (9.7) | 17 (6.3) |
| None at all | 22 (15.2) | 16 (12.9) | 38 (14.1) |
| Missing | 13 (9.0) | 13 (10.5) | 26 (9.7) |

^a^ Most survivors were admitted to hospital on the day of their stroke, however a small number of survivors receiving usual care were admitted a considerably longer period of time after the day of their stroke, explaining the increased mean and standard deviation in this arm.

^b^One intervention site, which recruited a large number of survivors, was unable to provide mRS or NIHSS data for any registered patients, explaining the large number of missing values in this arm.

**Table F: Baseline characteristics of participants in intervention sites split by intervention receipt**

|  | **Intervention not received**  **(n=59)** | **Intervention received**  **(n=86)** | **Total**  **(n=145)** |
| --- | --- | --- | --- |
| **Age**, years (s.d.) | 74 (12) | 71 (10) | 72 (11) |
| **Gender**, n female (%) | 27 (45.8) | 37 (43.0) | 64 (44.1) |
| **Ethnicity**, n (%) |  |  |  |
| White | 47 (79.7) | 68 (79.1) | 115 (79.3) |
| Other | 1 (1.7) | 1 (1.2%) | 2 (1.4) |
| Not stated | 11 (18.6) | 17 (19.8) | 28 (19.3) |
| **Marital** **status**, n (%) |  |  |  |
| Single | 2 (3.4) | 5 (5.8) | 7 (4.8) |
| Married/Living as married | 37 (62.7) | 52 (60.5) | 89 (61.4) |
| Separated/Divorced/Widowed | 18 (30.5) | 28 (32.6) | 46 (31.7) |
| Missing | 2 (3.4) | 1 (1.2) | 3 (2.1) |
| **Living arrangement,** n (%) |  |  |  |
| Living alone | 16 (27.1) | 24 (27.9) | 40 (27.6) |
| Living with another person | 41 (69.5) | 59 (68.6) | 100 (69.0) |
| Missing | 2 (3.4) | 3 (3.5) | 5 (3.4) |
| **Education level,** n (%) |  |  |  |
| None | 0 (0.0) | 2 (2.3) | 2 (1.4) |
| Primary School | 4 (6.8) | 1 (1.2) | 5 (3.4) |
| Secondary School | 29 (49.2) | 45 (52.3) | 74 (51.0) |
| Further / higher education | 26 (44.1) | 35 (40.7) | 61 (42.1) |
| Missing | 0 (0.0) | 3 (3.5) | 3 (2.1) |
| **Time since stroke , months (s.d.)** | 5.2 (0.77) | 5.4 (0.66) | 5.4 (0.71) |
| *Missing, n (%)* | *0 (0.0)* | *1 (1.2)* | *1 (0.7)* |
| **Level of language ability after stroke**, n (%) |  |  |  |
| Normal | 24 (40.7) | 23 (26.7) | 47 (32.4) |
| Dysphasia/ Dysarthria | 1 (1.7) | 7 (8.1) | 8 (5.5) |
| Not known | 34 (57.6) | 56 (65.1) | 90 (62.1) |
| **Length of hospital admission,** days (s.d.) | 8 (11) | 13 (21) | 11 (18) |
| *Missing, n (%)* | *1* | *3* | *4* |
| **Time between onset/awareness of stroke and hospital admission**, days (s.d.) | 0.2 (0.70) | 0.0 (0.93) | 0.1 (0.84) |
| *Missing, n (%)* | *1 (1.7)* | *2 (2.3)* | *3 (2.1)* |
| **mRS at discharge**, n (%) |  |  |  |
| 0 | 7 (11.9) | 4 (4.7) | 11 (7.6) |
| 1 | 15 (25.4) | 7 (8.1) | 22 (15.2) |
| 2 | 3 (5.1) | 4 (4.7) | 7 (4.8) |
| 3 | 4 (6.8) | 4 (4.7) | 8 (5.5) |
| 4 | 1 (1.7) | 0 (0.0) | 1 (0.7) |
| 5 | 0 (0.0) | 1 (1.2) | 1 (0.7) |
| Missing | 29 (49.2) | 66 (76.7) | 95 (65.5) |
| **NIHSS score at admission**, points (s.d.) | 5.2 (4.8) | 3.7 (4.1) | 4.5 (4.5) |
| *Missing, n (%)* | *27 (45.8)* | *61 (70.9)* | *88 (60.7)* |
| **WHODAS*** |  |  |  |
| **Simple score**, points (s.d.) | 25.6 (18.6) | 22.3 (17.8) | 23.7 (18.1) |
| *Missing, n (%)* | *12 (20.3)* | *16 (18.6)* | *28 (19.3)* |
| **Complex score**, points (s.d.) | 26.9 (20.3) | 24.3 (18.6) | 25.6 (19.3) |
| *Missing, n (%)* | *22 (37.3)* | *46 (53.5)* | *68 (46.9)* |
| **WEMWBS score^#^,** points (s.d.) | 48.4 (11.7) | 46.9 (11.6) | 47.5 (11.6) |
| *Missing, n (%)* | *0 (0.0)* | *0 (0.0)* | *0 (0.0)* |
| **PAM score^** , points (s.d.) | 58.6 (18.0) | 58.6 (17.6) | 58.6 (17.7) |
| *Missing, n (%)* | *0 (0.0)* | *2 (2.3)* | *2 (1.4)* |
| **PAM level (categorised PAM score)**, n (%) |  |  |  |
| (<=47.0) not believing activation important | 14 (23.7) | 22 (25.6) | 36 (24.8) |
| (47.1-55.1) a lack of knowledge and confidence to take action | 21 (35.6) | 24 (27.9) | 45 (31.0) |
| (55.2-67.0) beginning to take action | 9 (15.3) | 20 (23.3) | 29 (20.0) |
| (>=67.1) taking action | 15 (25.4) | 18 (20.9) | 33 (22.8) |
| Missing | 0 (0.0) | 2 (2.3) | 2 (1.4) |
| **LUNS (number of long-term unmet needs)**, n (%) |  |  |  |
| 0 | 9 (15.3) | 7 (8.1) | 16 (11.0) |
| 1-4 | 30 (50.8) | 39 (45.3) | 69 (47.6) |
| ≥5 | 20 (33.9) | 40 (46.5) | 60 (41.4) |
| **Social questions (how much support),** n (%) |  |  |  |
| ***Help around house when ill*** |  |  |  |
| A lot | 41 (69.5) | 55 (64.0) | 96 (66.2) |
| Some | 15 (25.4) | 17 (19.8) | 32 (22.1) |
| Not much | 2 (3.4) | 7 (8.1) | 9 (6.2) |
| None at all | 1 (1.7) | 7 (8.1) | 8 (5.5) |
| ***Help with heavy jobs*** |  |  |  |
| A lot | 42 (71.2) | 47 (54.7) | 89 (61.4) |
| Some | 11 (18.6) | 26 (30.2) | 37 (25.5) |
| Not much | 3 (5.1) | 7 (8.1) | 10 (6.9) |
| None at all | 3 (5.1) | 6 (7.0) | 9 (6.2) |
| ***Advice on important changes*** |  |  |  |
| A lot | 30 (50.8) | 27 (31.4) | 57 (39.3) |
| Some | 11 (18.6) | 23 (26.7) | 34 (23.4) |
| Not much | 4 (6.8) | 11 (12.8) | 15 (10.3) |
| None at all | 6 (10.2) | 14 (16.3) | 20 (13.8) |
| Missing | 8 (13.6) | 11 (12.8) | 19 (13.1) |
| ***Problems with spouse*** |  |  |  |
| A lot | 30 (50.8) | 28 (32.6) | 58 (40.0) |
| Some | 11 (18.6) | 22 (25.6) | 33 (22.8) |
| Not much | 2 (3.4) | 6 (7.0) | 8 (5.5) |
| None at all | 6 (10.2) | 12 (14.0) | 18 (12.4) |
| Missing | 10 (16.9) | 18 (20.9) | 28 (19.3) |
| ***Feeling depressed*** |  |  |  |
| A lot | 35 (59.3) | 33 (38.4) | 68 (46.9) |
| Some | 15 (25.4) | 30 (34.9) | 45 (31.0) |
| Not much | 4 (6.8) | 8 (9.3) | 12 (8.3) |
| None at all | 4 (6.8) | 9 (10.5) | 13 (9.0) |
| Missing | 1 (1.7) | 6 (7.0) | 7 (4.8) |
| ***Help caring for someone*** |  |  |  |
| A lot | 21 (35.6) | 19 (22.1) | 40 (27.6) |
| Some | 4 (6.8) | 12 (14.0) | 16 (11.0) |
| Not much | 3 (5.1) | 1 (1.2) | 4 (2.8) |
| None at all | 5 (8.5) | 19 (22.1) | 24 (16.6) |
| Missing | 26 (44.1) | 35 (40.7) | 61 (42.1) |
| ***Need someone to look after your home when away*** |  |  |  |
| A lot | 35 (59.3) | 36 (41.9) | 71 (49.0) |
| Some | 13 (22.0) | 21 (24.4) | 34 (23.4) |
| Not much | 1 (1.7) | 4 (4.7) | 5 (3.4) |
| None at all | 5 (8.5) | 17 (19.8%) | 22 (15.2) |
| Missing | 5 (8.5) | 8 (9.3) | 13 (9.0) |

Values reported are mean (s.d.) unless otherwise stated.

*higher score indicates higher level of disability; ^#^higher score indicates better state of wellbeing; ^higher score indicates higher level of activation

**Table G**: Description of usual care by site

| **Site** | **Recruited stroke survivors (n)** | **Stroke survivors offered a post-stroke review (n, % of recruited)** | **Stroke survivors seen/spoken to (n, % of offered)** | **No. of stroke survivor contacts (n, % of seen)** | | | **Average duration of contacts (mins), mean (SD)** |
| --- | --- | --- | --- | --- | --- | --- | --- |
|  |  |  |  | **1** | **2** | **≥3** |  |
| ***New Start sites*** | | | | | | | |
| 1 | 13 | N/A | N/A | N/A | N/A | N/A | N/A |
| 2 | 4 | 4 (100) | 3 (75.0) | - | - | - | - |
| 3 | 89 | N/A | N/A | N/A | N/A | N/A | N/A |
| 4 | 22 | 22 (100) | 10 (45.5) | 9 (90.0) | 1 (10.0) | 0 (0.0) | 21 (20)^a^ |
| 5 | 17 | 14 (82.4) | 11 (78.6) | 2 (18.2) | 2 (18.2) | 7 (6.4) | 63 (16) |
| *Total* | *145* | *40 (27.6)* | *24 (60.0)* | *11 (4.6)* | *3 (12.5)* | *7 (29.2)* | *44 (18)* |
| ***Control sites*** | | | | | | | |
| 6 | 42 | 41 (97.6) | 36 (87.8) | 36 (100) | 0 (0) | 0 (0) | 79 (23) |
| 7 | 10 | 10 (100) | 8 (80.0) | 8 (100) | 0 (0) | 0 (0) | 15 (0) |
| 8 | 29 | 28 (96.6) | 21 (75.0) | 14 (66.7) | 2 (9.5) | 5 (23.8) | 45 (.)^#^ |
| 9 | 28 | 26 (92.9) | 25 (96.2) | 22 (96.0) | 3 (4.0) | 0 (0) | 35 (11) |
| 10 | 15 | 3 (20.0) | 3 (100.0) | 1 (33.3) | 0 (0) | 2 (66.7) | 47 (6) |
| *Total* | *124* | *108 (87.1)* | *93 (86.1)* | *81 (87.1)* | *5 (5.4)* | *7 (7.5)* | *54 (27)* |

N/A - Delivering New Start only; - = missing data; ^a^n=5 with missing data; ^#^n=20 with missing data

**Table H: Facilitator unblinding by site**

| **Site** | **Recruited stroke**  **survivors (n)** | **First unblinding** | | **Second unblinding** |
| --- | --- | --- | --- | --- |
|  |  | **Informed by**  **stroke survivor** | **Other** | **Informed by**  **stroke survivor** |
| 1 | 13 | 1 (7.7%) | 0 ( 0.0%) | 0 ( 0.0%) |
| 2 | 4 | 0 ( 0.0%) | 0 ( 0.0%) | 0 ( 0.0%) |
| 3 | 89 | 10 (11.2%) | 1 (1.1%) | 0 ( 0.0%) |
| 4 | 22 | 1 (4.5%) | 0 ( 0.0%) | 1 (4.5%) |
| 5 | 17 | 0 ( 0.0%) | 0 ( 0.0%) | 0 ( 0.0%) |
| 6 | 42 | 0 ( 0.0%) | 0 ( 0.0%) | 0 ( 0.0%) |
| 7 | 10 | 0 ( 0.0%) | 0 ( 0.0%) | 0 ( 0.0%) |
| 8 | 29 | 0 ( 0.0%) | 0 ( 0.0%) | 0 ( 0.0%) |
| 9 | 28 | 0 ( 0.0%) | 0 ( 0.0%) | 0 ( 0.0%) |
| 10 | 15 | 0 ( 0.0%) | 0 ( 0.0%) | 0 ( 0.0%) |
| Total | 269 | 12 (4.5%) | 1 (0.4%) | 1 (0.4%) |

There were 14 reported occasions of New Start facilitator unblinding to their patients’ research participation, occurring across three sites and 13 patients. Twelve patients informed New Start facilitators of their trial participation (one of these informed the facilitator on two occasions) according to facilitator reports. One New Start facilitator was inadvertently informed of the trial participation of one patient after seeing a copy of their consent form on SystmOne. Most unblindings occurred within the first month post registration, an average of 20 days after registration.

**Table I:** Questionnaire completeness at all time points

|  | **Baseline** | | **3 months** | | **6 months** | | **9 months** | |
| --- | --- | --- | --- | --- | --- | --- | --- | --- |
|  | **New Start** | **Usual Care** | **New Start** | **Usual Care** | **New Start** | **Usual Care** | **New Start** | **Usual Care** |
| **Number of available participants** | 145 | 124 | 142 | 120 | 136 | 116 | 131 | 108 |
| **WHODAS** |  |  |  |  |  |  |  |  |
| **Questionnaire completion**, n (% of available participants) |  |  |  |  |  |  |  |  |
| Completed | 23 (15.9) | 25 (20.2) |  |  | 23 (16.9) | 25 (21.6) | 28 (21.4) | 20 (18.5) |
| Partially completed | 122 (84.1) | 97 (78.2) |  |  | 104 (76.5) | 76 (65.5) | 89 (67.9) | 71 (65.7) |
| Not completed | 0 (0.0) | 2 (1.6) |  |  | 9 (6.6) | 15 (12.9) | 14 (10.7) | 17 (15.7) |
| **Simple score,** n (% of partially completed) |  |  |  |  |  |  |  |  |
| Prorated | 94 (77.0) | 79 (81.4) |  |  | 80 (76.9) | 61 (80.3) | 73 (82.0) | 55 (77.5) |
| Score Missing | 28 (23.0) | 18 (18.6) |  |  | 24 (23.1) | 15 (19.7) | 16 (18.0) | 16 (22.5) |
| **Complex score,** n (% of partially completed) |  |  |  |  |  |  |  |  |
| Prorated | 54 (44.3) | 43 (44.3) |  |  | 48 (46.2) | 35 (46.1) | 44 (49.4) | 36 (50.7) |
| Score Missing | 68 (55.7) | 54 (55.7) |  |  | 56 (53.8) | 41 (53.9) | 45 (50.6) | 35 (49.3) |
| **Number of missing items** |  |  |  |  |  |  |  |  |
| Mean (s.d.) | 4.5 (4.00) | 4.2 (3.50) |  |  | 4.5 (4.44) | 4.7 (5.28) | 4.9 (6.14) | 4.2 (3.89) |
| **WEMWBS** |  |  |  |  |  |  |  |  |
| **Questionnaire completion**, n (% of available participants) |  |  |  |  |  |  |  |  |
| Completed | 135 (93.1) | 109 (87.9) |  |  | 106 (77.9) | 85 (73.3) | 104 (79.4) | 82 (75.9) |
| Partially completed | 10 (6.9) | 13 (10.5) |  |  | 22 (16.2) | 17 (14.7) | 16 (12.2) | 9 (8.3) |
| Not completed | 0 (0.0) | 2 (1.6) |  |  | 8 (5.9) | 14 (12.1) | 11 (8.4) | 17 (15.7) |
| **Score**, n (% of partially completed) |  |  |  |  |  |  |  |  |
| Prorated | 10 (100.0%) | 9 (69.2%) |  |  | 13 (59.1%) | 9 (52.9%) | 9 (56.3%) | 5 (55.6%) |
| Score Missing | 0 (0.0%) | 4 (30.8%) |  |  | 9 (40.9%) | 8 (47.1%) | 7 (43.8%) | 4 (44.4%) |
| **Number of missing items** |  |  |  |  |  |  |  |  |
| Mean (s.d.) | 0.1 (0.32) | 0.5 (2.28) |  |  | 1.0 (3.25) | 1.2 (3.65) | 0.8 (3.01) | 0.6 (2.61) |
| **PAM** |  |  |  |  |  |  |  |  |
| **Questionnaire completion**, n (% of available participants) |  |  |  |  |  |  |  |  |
| Completed | 132 (91.0) | 111 (89.5) | 116 (81.7) | 92 (76.7) | 104 (76.5%) | 85 (73.3%) |  |  |
| Partially completed | 13 (9.0) | 11 (8.9) | 20 (14.1) | 18 (15.0) | 25 (18.4%) | 17 (14.7%) |  |  |
| Not completed | 0 (0.0) | 2 (1.6) | 6 (4.2) | 10 (8.3) | 7 (5.1%) | 14 (12.1%) |  |  |
| **Score**, n (% of partially completed) |  |  |  |  |  |  |  |  |
| Prorated | 11 (84.6) | 10 (90.9) | 10 (50.0) | 12 (66.7) | 13 (52.0) | 9 (52.9) |  |  |
| Score Missing | 2 (15.4) | 1 (9.1) | 10 (50.0) | 6 (33.3) | 12 (48.0) | 8 (47.1) |  |  |
| **Number of missing items** |  |  |  |  |  |  |  |  |
| Mean (s.d.) | 0.5 (2.20) | 0.3 (1.41) | 1.0 (3.20) | 0.8 (2.87) | 1.5 (3.90) | 1.1 (3.36) |  |  |
| **LUNS** |  |  |  |  |  |  |  |  |
| **Questionnaire completion** n (% of available participants) |  |  |  |  |  |  |  |  |
| Completed | 117 (80.7) | 97 (78.2) |  |  |  |  | 86 (65.6) | 63 (58.3) |
| Partially completed | 28 (19.3) | 25 (20.2) |  |  |  |  | 34 (26.0) | 28 (25.9) |
| Not completed | 0 (0.0) | 2 (1.6) |  |  |  |  | 11 (8.4) | 17 (15.7) |
| **Number of missing items** |  |  |  |  |  |  |  |  |
| Mean (s.d.) | 0.4 (1.30) | 0.8 (2.87) |  |  |  |  | 0.9 (2.54) | 1.5 (4.28) |

**Table J:** Summary statistics of all outcome measures at various time points – patient-level

|  | **Baseline** | | | **3 months** | | | **6 months** | | | **9 months** | | |
| --- | --- | --- | --- | --- | --- | --- | --- | --- | --- | --- | --- | --- |
|  | **New**  **Start** | **Usual Care** | **Total** | **New Start** | **Usual Care** | **Total** | **New Start** | **Usual Care** | **Total** | **New Start** | **Usual Care** | **Total** |
| **WHODAS** |  |  |  |  |  |  |  |  |  |  |  |  |
| **Simple score,** points (s.d.) | 23.7 (18.1) | 26.2 (20.8) | 24.9 (19.4) |  |  |  | 20.6 (17.4) | 23.3 (20.4) | 21.8 (18.8) | 21.0 (18.2) | 22.0 (20.2) | 21.4 (19.0) |
| *Missing, n* | *28* | *20* | *48* |  |  |  | *41* | *38* | *79* | *43* | *49* | *92* |
| **Complex score,** points (s.d.) | 25.6 (19.3) | 26.9 (24.3) | 26.2 (21.7) |  |  |  | 22.0 (20.5) | 23.9 (24.2) | 22.9 (22.2) | 22.6 (20.1) | 23.7 (24.8) | 23.1 (22.2) |
| *Missing, n* | *68* | *56* | *124* |  |  |  | *74* | *64* | *138* | *73* | *68* | *141* |
| **WEMWBS score**, points (s.d.) | 47.5 (11.6) | 46.6 (12.6) | 47.1 (12.1) |  |  |  | 46.5 (11.4) | 46.0 (12.2) | 46.2 (11.7) | 46.8 (11.7) | 47.6 (11.7) | 47.1 (11.7) |
| *Missing, n* | *0* | *6* | *6* |  |  |  | *25* | *30* | *55* | *31* | *37* | *68* |
| **PAM** |  |  |  |  |  |  |  |  |  |  |  |  |
| **Continuous score,** points (s.d.) | 58.6 (17.7) | 56.7 (16.9) | 57.7 (17.4) | 55.0 (15.5) | 54.5 (15.1) | 54.8 (15.3) | 57.5 (18.0) | 56.4 (18.6) | 57.1 (18.2) |  |  |  |
| *Missing, n* | *2* | *3* | *5* | *19* | *22* | *41* | *30* | *37* | *67* |  |  |  |
| **Categorical score,** n (%) |  |  |  |  |  |  |  |  |  |  |  |  |
| (<=47.0) not believing activation important | 36 (24.8) | 38 (30.6) | 74 (27.5) | 34 (23.4) | 29 (23.4) | 63 (23.4) | 27 (18.6) | 25 (20.2) | 52 (19.3) |  |  |  |
| (47.1-55.1) a lack of knowledge and confidence to take action | 45 (31.0) | 32 (25.8) | 77 (28.6) | 42 (29.0) | 40 (32.3) | 82 (30.5) | 36 (24.8) | 25 (20.2) | 61 (22.7) |  |  |  |
| (55.2-67.0) beginning to take action | 29 (20.0) | 30 (24.2) | 59 (21.9) | 29 (20.0) | 17 (13.7) | 46 (17.1) | 28 (19.3) | 22 (17.7) | 50 (18.6) |  |  |  |
| (>=67.1) taking action | 33 (22.8) | 21 (16.9) | 54 (20.1) | 21 (14.5) | 16 (12.9) | 37 (13.8) | 24 (16.6) | 15 (12.1) | 39 (14.5) |  |  |  |
| Missing | 2 (1.4) | 3 (2.4) | 5 (1.9) | 19 (13.1) | 22 (17.7) | 41 (15.2) | 30 (20.7) | 37 (29.8) | 67 (24.9) |  |  |  |
| **LUNS (number of long-term unmet needs)** |  |  |  |  |  |  |  |  |  |  |  |  |
| **Count**, n (%) |  |  |  |  |  |  |  |  |  |  |  |  |
| 0 | 16 (11.0) | 19 (15.3) | 35 (13.0) |  |  |  |  |  |  | 33 (22.8) | 21 (16.9) | 54 (20.1) |
| 1-4 | 69 (47.6) | 62 (50.0) | 131 (48.7) |  |  |  |  |  |  | 65 (44.8) | 47 (37.9) | 112 (41.6) |
| ≥5 | 60 (41.4) | 41 (33.1) | 101 (37.5) |  |  |  |  |  |  | 23 (15.9) | 23 (18.5) | 46 (17.1) |
| Missing | 0 (0.0) | 2 (1.6) | 2 (0.7) |  |  |  |  |  |  | 24 (16.6) | 33 (26.6) | 57 (21.2) |
| **Mean (s.d.)** | 4.1 (3.2) | 3.8 (3.6) | 4.0 (3.4) |  |  |  |  |  |  | 2.7 (3.2) | 3.1 (3.1) | 2.9 (3.2) |
| *Missing, n* | *0* | *2* | *2* |  |  |  |  |  |  | *24* | *33* | *57* |

Values reported are mean (s.d.) unless otherwise stated.

**
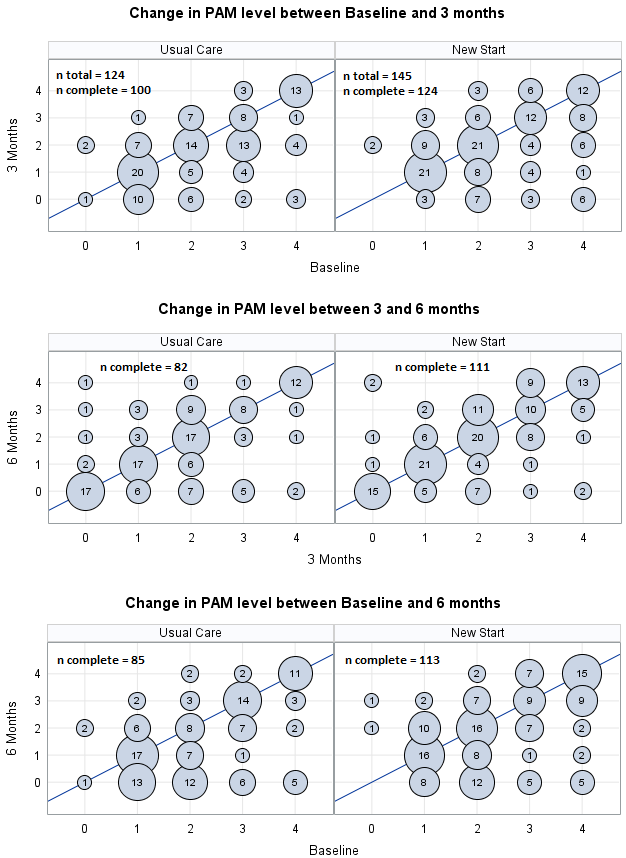
**

**Figure 2 - Change in Patient Activation Measure (PAM) category between Baseline, 3 and 6 month time points**

**Table K: ICC estimates for patient reported outcomes**

|  | **Time point** | **Number of non-missing observations** | **Estimated coefficient of reliability, ICC (95% CI)** | **Estimated coefficient of within-subject variance (95% CI)** |
| --- | --- | --- | --- | --- |
| **WHODAS simple score** |  |  |  |  |
|  | Baseline | 217 | 0.02 (0.00, 0.23) | 0.77 (0.65, 0.90) |
|  | 6 months | 188 | 0.00 (., .) | 0.86 (0.73, 1.00) |
|  | 9 months | 175 | 0.00 (., .) | 0.88 (0.74, 1.04) |
| **WHODAS complex score** |  |  |  |  |
|  | Baseline | 142 | 0.01 (0.00, 0.95) | 0.81 (0.67, 0.97) |
|  | 6 months | 130 | 0.00 (., .) | 0.94 (0.77, 1.15) |
|  | 9 months | 127 | 0.00 (., .) | 0.93 (0.76, 1.14) |
| **WEMWBS score** |  |  |  |  |
|  | Baseline | 259 | 0.00 (., .) | 0.26 (0.23, 0.28) |
|  | 6 months | 212 | 0.00 (., .) | 0.25 (0.23, 0.28) |
|  | 9 months | 199 | 0.00 (., .) | 0.25 (0.22, 0.28) |

**Table L:** Hospitalisation and institutionalisation reported by stroke survivors by arm

|  | **Baseline** | | **3 months** | | **6 months** | | **9 months** | |
| --- | --- | --- | --- | --- | --- | --- | --- | --- |
|  | **New Start**  **(n=145)** | **Usual Care**  **(n=124)** | **New Start**  **(n=145)** | **Usual Care**  **(n=124)** | **New Start**  **(n=145)** | **Usual Care**  **(n=124)** | **New Start**  **(n=145)** | **Usual Care**  **(n=124)** |
| **Number of completed health economics booklets**, n (% of recruited) | 142 (97.9) | 116 (93.5) | 132 (91.0) | 105 (84.7) | 124 (85.5) | 97 (78.2) | 116 (80.0) | 86 (69.4) |
| **Hospital inpatient stay (Y), n (%*)** | 15 (10.6) | 20 (17.2) | 13 (9.8) | 14 (13.3) | 13 (10.5) | 8 (8.2) | 10 (8.6) | 8 (9.3) |
| **Number of days in hospital** | 11.0 (18.3) | 12.7 (12.0) | 8.5 (10.6) | 6.4 (5.3) | 11.5 (19.9) | 8.7 (10.1) | 6.9 (5.3) | 2.9 (1.7) |
| *Missing, n* | *4* | *4* | *2* | *2* | *2* | *1* | *3* | *1* |
| **Total number of visits during last 3 months** | 1.7 (1.0) | 2.0 (1.5) | 1.4 (0.8) | 1.6 (1.5) | 1.2 (0.4) | 1.3 (0.5) | 1.8 (1.7) | 1.0 (0.0) |
| *Missing, n* | *9* | *7* | *6* | *7* | *7* | *4* | *6* | *3* |
| **Hospital A&E department visit (Y), n (%*)** | 15 (10.6) | 16 (13.8) | 19 (14.4) | 11 (10.5) | 12 (9.7) | 7 (7.2) | 17 (14.7) | 5 (5.8) |
| **Total number of visits during last 3 months** | 1.5 (1.6) | 1.6 (0.7) | 1.2 (0.4) | 1.4 (0.5) | 1.7 (2.2) | 1.5 (0.8) | 1.2 (0.4) | 2.0 (0.0) |
| *Missing, n* | *5* | *4* | *6* | *6* | *2* | *1* | *6* | *3* |
| **Nursing/residential home stay (Y), n(%*)** | 3 (2.1%) | 0 (0.0%) | 0 (0.0%) | 1 (1.0%) | 0 (0.0%) | 2 (2.1%) | 0 (0.0%) | 0 (0.0%) |
| **Number of days in nursing/residential home** | 29.0 (.) |  |  | 90.0 (.) |  | 14.0 (.) |  |  |
| **Total number of visits during last 3 months** | . (.) |  |  | . (.) |  | . (.) |  |  |
| *Missing, n* | *3* |  |  | *1* |  | *2* |  |  |

*% of total completed booklets.

Values reported are mean (s.d.) unless otherwise stated.

**Table M: Recruitment progression criteria**

| **Site** | **Total number**  **recruited** | **Screening length**  **(months)** | **Average monthly**  **recruitment** | **6m pro-**  **rated**  **recruitment** |
| --- | --- | --- | --- | --- |
| 3 | 89 | 5.1 | 17.5 | 105 |
| 6 | 42 | 8.1 | 5.2 | 31.1 |
| 4 | 22 | 4.6 | 5 | 28.7 |
| 8 | 29 | 7.4 | 3.9 | 23.5 |
| 9 | 28 | 8 | 3.5 | 21.1 |
| 1 | 13 | 5.3 | 2.5 | 14.7 |
| 10 | 15 | 6.5 | 2.3 | 13.9 |
| 5 | 17 | 8.1 | 2.1 | 12.6 |
| 7 | 10 | 5.9 | 1.7 | 10.2 |
| 2 | 4 | 7.9 | 0.5 | 3 |
| **Total** | **269** | **66.9** | **4.0** | **24.1** |

**Table N: Follow up progression criteria (follow-up at 9 months)**

| **Treatment allocation** | **N** | **Booklets returned** | **Follow up rate** |
| --- | --- | --- | --- |
| Usual Care | 124 | 94 | 75.8% |
| New Start | 145 | 122 | 84.1% |
| **Total** | **269** | **216** | **80.3%** |

**Table O: Baseline demographic characteristics and measures of carers by treatment arm**

|  | **New Start**  **(n=46)** | **Usual Care**  **(n=39)** | **Total**  **(n=85)** |
| --- | --- | --- | --- |
| **Age**, years (s.d.) | 68 (10.7) | 63 (13.0) | 66 (12.0) |
| *Missing, n* | *1* | *1* | *2* |
| **Gender**, n female (%) | 29 (63.0) | 28 (71.8) | 57 (67.1) |
| **Who caring for,** n (%) |  |  |  |
| Spouse / partner | 37 (80.4) | 23 (59.0) | 60 (70.6) |
| Parent | 5 (10.9) | 7 (17.9) | 12 (14.1) |
| Child (including in-law, step) | 1 (2.2) | 8 (20.5) | 9 (10.6) |
| Other relative/Friend | 3 (6.5) | 1 (2.6) | 2 (2.4) |
| **CBS**, points (s.d.) |  |  |  |
| **Total score** | 45.6 (15.3) | 48.7 (15.3) | 47.0 (15.3) |
| *Missing, n* | *0* | *1* | *1* |
| ***General strain sub-score*** | 17.9 (6.3) | 20.1 (7.0) | 18.9 (6.7) |
| *Missing, n* | *0* | *1* | *1* |
| ***Isolation sub-score*** | 6.9 (2.5) | 7.3 (2.5) | 7.1 (2.5) |
| *Missing, n* | *0* | *3* | *3* |
| ***Disappointment sub-score*** | 10.8 (4.3) 0 | 11.3 (4.3) 1 | 11.0 (4.3) |
| *Missing, n* | *0* | *1* | *1* |
| ***Emotional involvement sub-score*** | 4.9 (2.3) | 4.8 (1.7) | 4.8 (2.0) |
| *Missing, n* | *0* | *1* | *1* |
| ***Environment sub-score*** | 5.0 (2.1) | 5.3 (2.0) | 5.1 (2.0) |
| *Missing, n* | *2* | *1* | *3* |

Values reported are mean (s.d.) unless otherwise stated. CBS = Caregiver Burden Scale

**Table P - Caregiver Burden Scale (CBS) questionnaire scores for all time points**

| **Total CBS score** | **New Start**  **(n=46)** | **Usual Care**  **(n=39)** |
| --- | --- | --- |
| **Baseline**  Mean (SD)  *Missing, n* | 45.6 (15.3)  *0* | 48.7 (15.3)  *1* |
| **3 months**  Mean (SD)  *Missing, n* | 47.7 (14.2)  *15* | 48.7 (14.8)  *19* |
| **6 months**  Mean (SD)  *Missing, n* | 46.8 (14.5)  *10* | 43.7 (14.2)  *14* |
| **9 months**  Mean (SD)  *Missing, n* | 48.2 (15.7)  *13* | 44.9 (16.1)  *15* |

| **TIDieR item** | **Description** |
| --- | --- |
| **1. Brief name** | *New Start*: a facilitated open process, including components of problem-solving and self-management, to enable a stroke survivor to address their identified needs. |
| **2. Why** | Goal: to improve stroke survivors’ quality of life by addressing unmet needs and increasing participation.  Rationale: poor longer-term outcome for patients and their carers after stroke; Stroke survivors often have a number of unmet needs but few means of addressing these, especially because healthcare provision reduces over time. Self-management approaches can enable individuals with long-term conditions to become active in managing their health, resulting in improved quality of life, and enhancing participation. there is a national guideline recommendation for a 6-month review of health and social care needs but no evidence-based intervention. Prior qualitative work identified myriad problems to address. Intervention activities should result in “Action taken by stroke survivor to address unmet needs; Actions by supporters; Actions by health professionals; Reframing of capabilities and circumstances by stroke survivor and/or supporter” (Hardicre et al., 2018) |
| **3. What (materials)** | “Materials to support needs assessment, self-management, goal-setting and action-planning, as well as the provision of useable information (the ‘priming tool’ and ‘New Start Guide’)” (Hardicre et al., 2018); patient activity records. |
| **4. What (procedures)** | “The New Start intervention will be offered to all stroke survivors within the stroke services allocated to the intervention arm and who are approximately 6 months post-stroke.” (Hardicre et al., 2018)  The stroke survivor is invited by letter or telephone call to receive a six-month review.  “The New Start intervention consists of an initial face-to-face meeting, at the stroke survivor’s home or in clinic, with a trained facilitator. It seeks to help stroke survivors identify any unmet needs they may have and then to work with them to address these needs. A leaflet providing a list of common problems faced by stroke survivors (termed a priming tool) will be sent out with an appointment letter in advance to highlight potential topics for discussion (unmet needs) and it invites the survivor to add their own. At the meeting, these issues and needs will be discussed (whether noted on the priming tool or not) and a supported self-management approach introduced” (Hardicre et al., 2018) incorporating “prioritising needs, action planning, goal setting, and reviewing as well as self-management” (Forster et al., 2018) with open-ended follow-up. |
| **5. Who provided** | A trained facilitator identified by the stroke service from their existing staff. At least two facilitators were identified and appointed by sites.  “New Start facilitators will have experience in one of the following roles: nurse, physiotherapist, occupational therapist, health and well-being practitioner, or will have other allied health professional training, and will have stroke-specific knowledge or training.”  Prior to training, facilitators watched a pre-training lecture (video) and completed an assessment. “Intervention training comprised an initial 2-day training session and two follow-up sessions. Facilitators then had the opportunity to practise intervention delivery before the trial commenced and were asked to complete two structured reflective reports focusing on New Start delivery during this practice phase.” (Forster et al., 2021) Materials, professional support and peer-support were available. Training was “delivered in collaboration with the central research team and Enabling Self-Care, the latter led by an independent specialist physiotherapist and consultant clinical health psychologist, both with years of experience in providing training and support in self-management knowledge and skills to health and social care professionals.” (Forster et al., 2021)  “Facilitators were appraised regarding their competency in delivering the New Start intervention.” (Forster et al., 2021) |
| **6. How** | Each service developed their own procedures for approaching stroke survivors (e.g. appointment letter, telephone call).  “one-to-one meetings that are guided by principles of patient centredness, empowerment for patients and carers, and open availability for feedback.” (Forster et al., 2018) The initial meeting was face-to-face.  “The process involves facilitated action-planning, goal-setting, and review, all of which are supported by New Start materials, a set of worksheets developed during previous phases of the LoTS2Care programme. Booklets containing information about stroke and useful contacts are also available and can be provided to stroke survivors as appropriate.” (Hardicre et al., 2018) Intervention activities include “Generating and maintaining understanding and a positive relationship between the facilitator and survivor; Active listening and Socratic questioning; Identifying unmet needs; Mapping existing social support networks; Survivor-directed (& facilitator supported) problem-solving (process); Prompting to action; Supporting action; Referrals to health and social care professionals” (Hardicre et al., 2018). “At each stage, participants are encouraged to see those in their social network as resources to help in this process.” (Hardicre et al., 2018)  Open-ended follow-up as appropriate including optional visits, phone calls, emails, etc. |
| **7. Where** | “at the stroke survivor’s home or in clinic” (Hardicre et al., 2018) |
| **8. When and how much** | Delivered to stroke survivors and (optionally) their carers approximately 6 months post-stroke  “Stroke survivors can have as many meetings with the facilitators as required. It is anticipated that most stroke survivors will have at least three visits and support may also be provided via other means (e.g. by phone or email).” (Hardicre et al., 2018) |
| **9. Tailoring** | Personalised based on identified needs, goals and resources. Open-ended follow-up. |
| **10. Modifications** | No modifications were made by the trial team to the New Start intervention |
| **11. How well (planned)** | “The New Start facilitators will be assessed for competency in the delivery of the New Start intervention, through review of patient activity records, reflective reports, interviews and observation, approximately 16 weeks after completing the initial training course” (Forster et al., 2018)  “Compliance with the New Start intervention will be monitored throughout the trial via observations and regular collection of activity records to assess adherence, to understand whether the New Start facilitators deliver the intervention in accordance with training and ‘as intended’.” (Forster et al., 2018)  “non-participant observation of […] intervention delivery. Interviews with stroke survivors, facilitators and other relevant staff (including administrators and managerial staff) will be undertaken. Qualitative data from interview transcripts, facilitator reflections and observational field notes will be analysed thematically alongside numerical data documenting intervention delivery collected as part of the trial.” (Hardicre et al., 2018) |
| **12. How well (actual)** | Fifteen facilitators were identified to deliver New Start across five sites (range 2-4 facilitators per site). Most facilitators attended the initial training session (n=14) and follow-up session (n=13). Face-to-face training was provided at a later date to one facilitator who had not attended the initial session. All facilitators were assessed as competent in intervention delivery based on predefined criteria following interviews and review of reflective reports and patient activity records.  In keeping with the cluster trial design, all stroke survivors at intervention sites were offered the intervention, not all of whom consented to outcome data collection. Across all intervention sites, at least 541 stroke survivors were contacted regarding New Start and 69.7% (n=377) went on to receive at least one session. Among trial participants, 138 out of 145 (95.2%) stroke survivors were contacted regarding New Start and 86 of these (59.3%) went on to receive at least one New Start session. These participants attended an average of 1.14 meetings, each lasting approximately 1 hour (see Table 4).  Results of the process evaluation are available in Forster et al., 2021. |


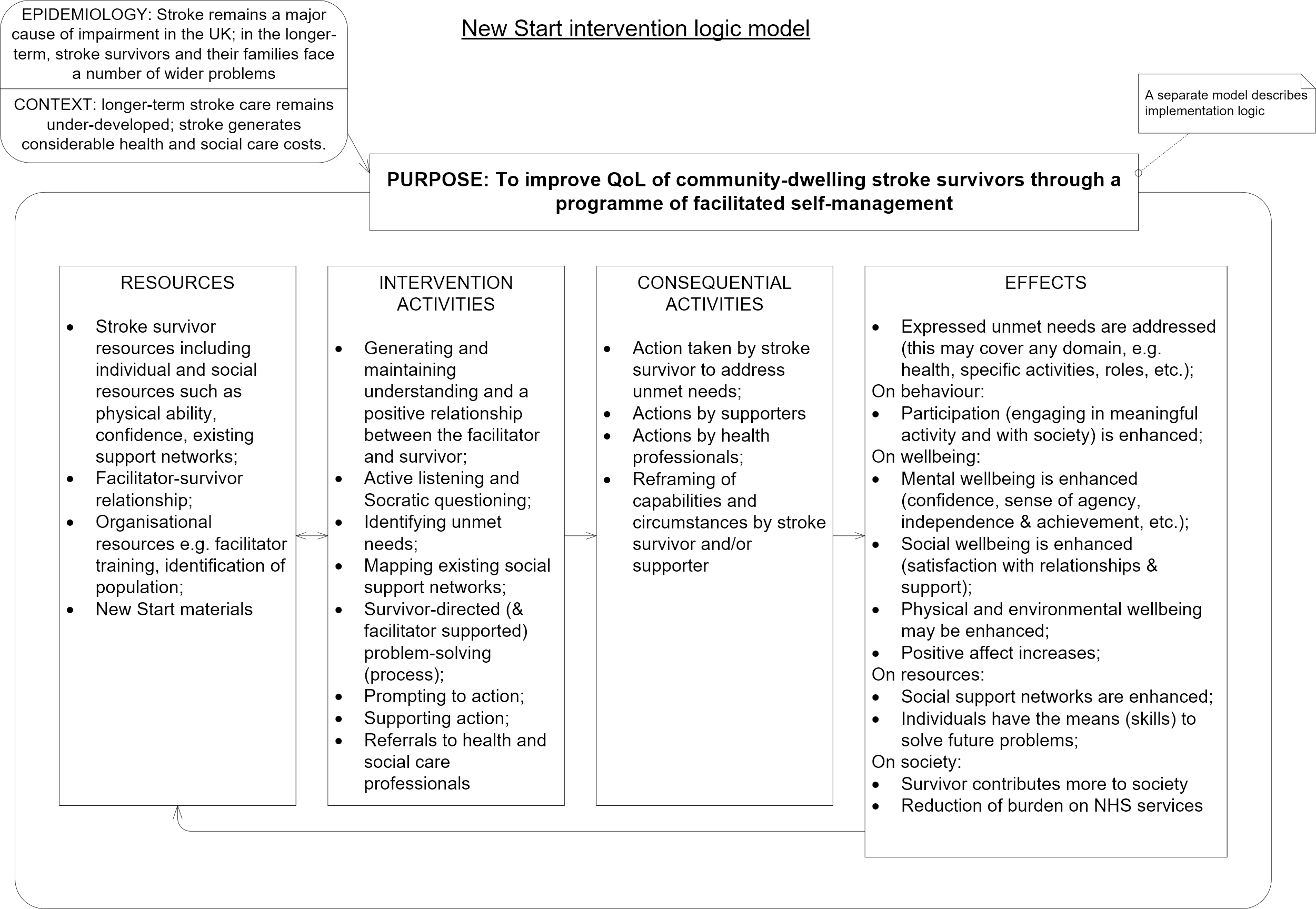


Reproduced from Hardicre et al., 2018

# References

Forster A, Hartley S, Barnard L, Ozer S, Hardicre N, Crocker T, *et al*. An intervention to support stroke survivors and their carers in the longer term (LoTS2Care): study protocol for a cluster randomised controlled feasibility trial. *Trials*. **2018**;19:317. <https://doi.org/10.1186/s13063-018-2669-5>

Forster A, Ozer S, Crocker TF, House A, Hewison J, Roberts E, *et al.* Longer-term health and social care strategies for stroke survivors and their carers: the LoTS2Care research programme including cluster feasibility RCT. *Programme Grants Appl Res.* **2021**;9(3):pgfar09030. <https://doi.org/10.3310/pgfar09030>

Hardicre NK, Crocker TF, Wright A, Burton LJ, Ozer S, Atkinson R, *et al*. An intervention to support stroke survivors and their carers in the longer term (LoTS2Care): study protocol for the process evaluation of a cluster randomised controlled feasibility trial. *Trials.* **2018**;19:368. <https://doi.org/10.1186/s13063-018-2683-7>

Hoffmann TC, Glasziou PP, Boutron I, Milne R, Perera R, Moher D, *et al*. Better reporting of interventions: template for intervention description and replication (TIDieR) checklist and guide. *BMJ*. **2014**;348:g1687. <https://doi.org/10.1136/bmj.g1687>
